# Development and validation of a machine learning-based diagnostic model for identifying nonneutropenic invasive pulmonary aspergillosis in suspected patients: a multicenter cohort study

**DOI:** 10.1128/spectrum.00607-25

**Published:** 2025-05-22

**Authors:** Xinyu Wang, Yajie Lu, Chao Sun, Huanhuan Zhong, Yuchen Cai, Min Cao, Xuefan Cui, Wenkui Sun, Li Wang, Xin Lu, Cheng Chen, Yanbin Chen, Chunlai Feng, Yujian Tao, Jun Zhou, Jiaxin Shi, Guoer Ma, Yuanqin Li, Xin Su

**Affiliations:** 1 Department of Respiratory and Critical Care Medicine, Nanjing Drum Tower Hospital, Affiliated Hospital of Medical School, Nanjing University, Nanjing, China; 2 Department of Respiratory and Critical Care Medicine, Jinling Hospital, Affiliated Hospital of Medical School, Nanjing University, Nanjing, China; 3 Department of Respiratory and Critical Care Medicine, The Second Affiliated Hospital of Soochow University, Suzhou, China; 4 Department of Respiratory and Critical Care Medicine, Jiangsu Province Hospital, The First Affiliated Hospital of Nanjing Medical University, Nanjing, China; 5 Department of Respiratory and Critical Care Medicine, Nanjing First Hospital 385685 , Nanjing, Jiangsu, China; 6 Department of Respiratory and Critical Care Medicine, The Second Affiliated Hospital of Nanjing University of Chinese Medicine 531909, Nanjing, Jiangsu, China; 7 Department of Respiratory and Critical Care Medicine, Nanjing Jiangning Hospital 579164, Nanjing, Jiangsu, China; 8 Department of Respiratory and Critical Care Medicine, The First Affiliated Hospital of Soochow University, Suzhou, China; 9 Department of Respiratory and Critical Care Medicine, Changzhou First People’s Hospital, Changzhou, China; 10 Department of Respiratory and Critical Care Medicine, Affiliated Hospital of Yangzhou University 632468, Yangzhou, Jiangsu, China; 11 Department of Respiratory and Critical Care Medicine, The First People’s Hospital of Lianyungang, Lianyungang, China; 12 Department of Respiratory and Critical Care Medicine, Affiliated Hospital of Jiangsu University 191612, Zhenjiang, Jiangsu, China; 13 Department of Respiratory and Critical Care Medicine, Affiliated Hospital of Xuzhou Medical University 117910 , Xuzhou, Jiangsu, China; University of Debrecen, Debrecen, Hungary

**Keywords:** nonneutropenic invasive pulmonary aspergillosis, machine learning, diagnostic model, weighted ensemble, *Aspergillus*-specific IgG, plasma pentraxin 3

## Abstract

This study aims to develop and validate an optimized diagnostic model for nonneutropenic invasive pulmonary aspergillosis (IPA) among suspected cases. A cohort of 344 nonneutropenic suspected cases from 13 medical centers (August 2020 to February 2024) was analyzed. The cohort was divided into a training data set (70%) and a testing data set (30%) using stratified sampling based on the IPA diagnosis. Three machine learning models (a regularized logistic regression model, a support vector machine model, and a weighted ensemble model) were developed. SHapley Additive explanation (SHAP) method was used for model interpretation. Six predictor variables were finally selected: sputum *Aspergillus* culture, *Aspergillus*-specific IgG, imaging feature of cavity, serum galactomannan, critical condition, and plasma pentraxin 3. The weighted ensemble model, exhibiting the significantly higher specificity of 95.1% in internal cross-validation and 95.7% in testing among the three models, was selected as the optimal prediction model despite comparable discrimination capacity, calibration ability, and clinical applicability across all models. The risk score derived from SHAP values showed a highly significant correlation with the predicted probability of the weighted ensemble model (Spearman *ρ* = 0.974), achieving an area under the curve of 0.857 in internal cross-validation and 0.871 in external testing. Using the optimal cut-off value of 3, the risk score demonstrated sensitivity (68.8%) and specificity (87.5%) comparable to those of bronchoalveolar lavage fluid galactomannan (cut-off = 1.0). The diagnostic model and risk score could assist in identifying nonneutropenic IPA from suspected cases independently of invasive procedures, thereby enhancing clinical applicability.

**IMPORTANCE:**

Although clinicians can screen out suspected cases through medical history inquiries, the diagnosis of nonneutropenic invasive pulmonary aspergillosis (IPA) from suspected cases remains a significant challenge. The study developed a novel diagnostic framework by integrating clinical parameters, imaging features, and laboratory biomarkers using machine learning techniques. The risk score, derived from SHapley Additive explanation values, exhibited a highly significant correlation with the predicted probability of the weighted ensemble model, demonstrating robust discrimination capacity and generalizability. The diagnostic model and risk score could assist in identifying nonneutropenic IPA from suspected cases independently of invasive procedures, thereby enhancing clinical applicability.

## INTRODUCTION

Invasive pulmonary aspergillosis (IPA) is a severe fungal infection frequently observed in severely immunocompromised individuals, particularly those suffering from neutropenia (1, 2). Previous studies and diagnostic guideline on IPA have primarily focused on these population. However, IPA is increasingly reported in nonneutropenic patients, such as those admitted to the intensive care unit (ICU), and individuals with chronic obstructive pulmonary disease (COPD), solid organ tumors, diabetes mellitus, or prolonged use of immunosuppressants (3–6).

The diagnosis of IPA relies on a combination of host factor, clinical feature, and mycological evidence (7). Clinicians can identify suspected nonneutropenic IPA patients, as they often present with host factors, abnormal chest imaging features, and an ineffective response to empirical anti-infective treatment. However, identifying IPA from suspected cases remains a challenge. Patients with nonneutropenic IPA often exhibit high heterogeneity due to varied baseline comorbidities and risk conditions (8, 9). The clinical symptoms and imaging features can be hardly distinguished from underlying diseases, leading to diagnostic delays and high mortality rates (10, 11). Furthermore, the low sensitivity of *Aspergillus* culture from respiratory samples complicates diagnosis, and the positive result is still difficult to distinguish from colonization (12). Our previous study demonstrated that *Aspergillus*-specific IgG exhibited a sensitivity of 59.6% at a cut-off of 80 AU/mL, higher than serum galactomannan (GM) test and comparable to bronchoalveolar lavage fluid (BALF) GM test (both with a cut-off value of 1.0) (13). However, the lower specificity (77% vs 96.3% and 91.2%) indicated that a comprehensive diagnosis still requires integrating additional clinical evidence beyond a single parameter.

To address current diagnostic challenges associated with nonneutropenic IPA, we conducted a cohort study and developed a machine learning-based diagnostic model (14) that integrated available evidence beyond typical parameters, providing an easy-to-use approach to accurately identify nonneutropenic IPA from suspected cases.

## MATERIALS AND METHODS

### Study design and population

The multicenter cohort study adhered to the clinical prediction model development guideline (15) and the TRIPOD + AI (Transparent Reporting of a Multivariable Prediction Model for Individual Prognosis or Diagnosis +Artificial Intelligence) statement (16).

This was a prospective, multicenter, observational cohort study, with suspected nonneutropenic IPA cases recruited from 13 medical centers in Jiangsu Province, including Nanjing Drum Tower Hospital, Nanjing Jinling Hospital, Jiangsu Province Hospital, Nanjing First Hospital, Nanjing Jiangning Hospital, The First Affiliated Hospital of Soochow University, The Second Affiliated Hospital of Soochow University, Changzhou First People’s Hospital, Affiliated Hospital of Yangzhou University, The First People’s Hospital of Lianyungang, Affiliated Hospital of Jiangsu University, The Second Affiliated Hospital of Nanjing University of Chinese Medicine, and Affiliated Hospital of Xuzhou Medical University from August 2020 to February 2024. All cases reached a final definite diagnosis of either IPA or Non-IPA.

Inclusion criteria for suspected nonneutropenic IPA (*n* = 652) included: (i) peripheral blood neutrophil count >0.5 × 10^9^ /L; (ii) potential risk factors for IPA, such as lung structural lesions, solid organ tumors, diabetes mellitus, admission to ICU, and prolonged use of immunosuppressants; (iii) new appeared chest computed tomography (CT) lesions, such as halo sign, cavity, nodule, and pleural effusion; (iv) poor response to empirical anti-infective therapy. Exclusion criteria included: (a) patients diagnosed with chronic pulmonary aspergillosis (*n* = 26), allergic bronchopulmonary aspergillosis (*n* = 9) or without a final definite diagnosis (*n* = 6); (ii) patients previously treated with antifungals against *Aspergillus* (*n* = 41); (iii) patients without baseline clinical information (*n* = 46); (iv) cases with missing *Aspergillus*-specific IgG values (*n* = 177). Additionally, the study concurrently excluded three patients with positive *Aspergillus* culture in respiratory tract samples but without clinical manifestations of invasive infection and other supporting evidence. Such cases conformed to the criteria of *Aspergillus* colonization rather than active infection. After exclusions, 344 patients were included in the current analysis. Taking into account the considerable number of cases with missing *A*spergillus-specific IgG and the potential selection bias, we carried out an additional sensitivity analysis after performing multiple imputations for the missing values.

The study adhered to the declaration of Helsinki and international standards for clinical trial registration, registered at the Chinese Clinical Trial Registry (ChiCTR) on 22 October 2020 (ChiCTR2000039235).

### Diagnosis of IPA

The diagnosis of IPA is mainly based on the guidelines of the European Organization for Research and Treatment of Cancer and the Mycoses Study Group Education and Research Consortium (EORTC/MSGERC) updated in 2020 (7). The host factors were consistent with the inclusion criteria for suspected nonneutropenic IPA (2). Clinical features of nonneutropenic IPA are often more varied and lack specificity (11), with chest CT abnormalities including, but not limited to, dense lesions (with or without halo sign), air crescent sign, cavities, and consolidation. And the diagnosis of patients with COPD or critical condition (admission to ICU, mechanical ventilation, severe pneumonia, sepsis/septic shock, or acute respiratory distress syndrome) also considered Bulpa criteria (17) and Invasive Fungal Diseases in Adult Patients in Intensive Care Unit (FUNDICU) 2024 consensus definitions (3). The final diagnosis was reconfirmed by the response to antifungal therapy.

### Candidate predictor variables and data collection

When establishing the model, all continuous variables were transformed into categorical variables. The potential predictor variables were categorized as follows: (i) demographic information: age (<65 years or ≥65 years), gender, body mass index (<18.5 kg/m^2^, 18.5–24.0 kg/m^2^, or ≥24.0 kg/m^2^), and smoking; (ii) host factors (2): critical condition, chronic lung structural lesions (COPD, lung cancer, bronchiectasis, tuberculosis, or interstitial lung disease), solid organ transplantation, solid organ tumor (except lung cancer), diabetes, chronic kidney disease, hepatopathy, autoimmune diseases, systemic application of glucocorticoids, and application of immunosuppressants; (iii) symptoms and imaging features (12); (iv) laboratory test: sputum *Aspergillus* culture (7), serum GM (<0.5, 0.5–1.0, or ≥1.0) (7), *Aspergillus*-specific IgG (<80 AU/mL or ≥80 AU/mL) (13), C-reactive protein (<10 mg/L or ≥10 mg/L), and procalcitonin (<0.5 ng/mL or ≥0.5 ng/mL). In the absence of a recognized cut-off value, the optimal cut-off value of plasma Pentraxin 3 (PTX3) was determined to be 4.4 ng/mL through receiver operating characteristic (ROC) curve analysis (<4.4 ng/mL or ≥4.4 ng/mL) (18, 19) (Fig. S1).

### Model training, evaluation, and application

The cohort was divided into a training data set (70%) and a testing data set (30%) using stratified sampling based on the IPA diagnosis. Missing values were imputed using multiple imputation based on the chained equations (MICE package in R) to reduce bias and improve statistical power (20). Moreover, we applied inverse probability weighting to the gender during model training to mitigate gender imbalance.

The variable selection process followed a three-phase approach: initial screening via univariate analysis with Bonferroni correction (adjusted significance threshold *α*/*k*, where *k* = number of variables) to reduce false positives; subsequent optimization using bidirectional stepwise regression guided by the Bayesian Information Criterion (BIC) to balance model complexity and predictive accuracy; and final validation through Bootstrap resampling iterations, retaining variables consistently selected in ≥80% of subsamples to ensure robustness against data variability. Spearman correlation (applicable for non-normal or ordinal variables) was used to assess correlations among selected variables, with multicollinearity indicated by a correlation coefficient >0.7.

After variable selection, a regularized logistic regression (RLR) model and a support vector machine (SVM) model with the radial basis function (RBF) kernel were developed. The RLR model was optimized over a hyperparameter grid covering Least Absolute Shrinkage and Selection Operator (LASSO), Ridge, and Elastic Net adopted (21), with alpha spanning 0–1, and regularization strength lambda spanning 10^−4^ to 1. For the SVM model, we searched the kernel parameter sigma and the penalty parameter C. Additionally, a weighted ensemble model was generated based on area under the curve (AUC) values of the two models, with weights assigned to each (22, 23). Model validation and parameter optimization were accomplished through fivefold, three-repeated cross-validation.

Model performance was evaluated on discrimination, calibration, and clinical applicability. The best-performing model was interpreted via SHapley Additive exPlanation (SHAP), and a risk score table was established for stratification. The correlation between the total risk score and the predicted probability was validated by Spearman analysis, confirming that the scoring system could effectively mirror the model predictions. The efficacy of the risk score for diagnosing IPA was determined through ROC analysis, and the optimal cut-off value for risk stratification was acquired. Sensitivity and subgroup analyses verified result robustness, and diagnostic performance of the risk score was compared with current guideline-recommended indicators.

For a detailed methods description, please refer to the Supplementary Methods. All statistical analyses were done with the software R (version 4.2.1). Continuous variables were summarized using mean ± standard deviation (normal distribution) or median (Q1, Q3) (non-normal distribution), while categorical variables were presented as frequency (*n*) and proportion (%). The statistical significance was determined at a *P* value < 0.05.

## RESULTS

### Study population and diagnostic performance of mycologic evidence

**Fig 1:**
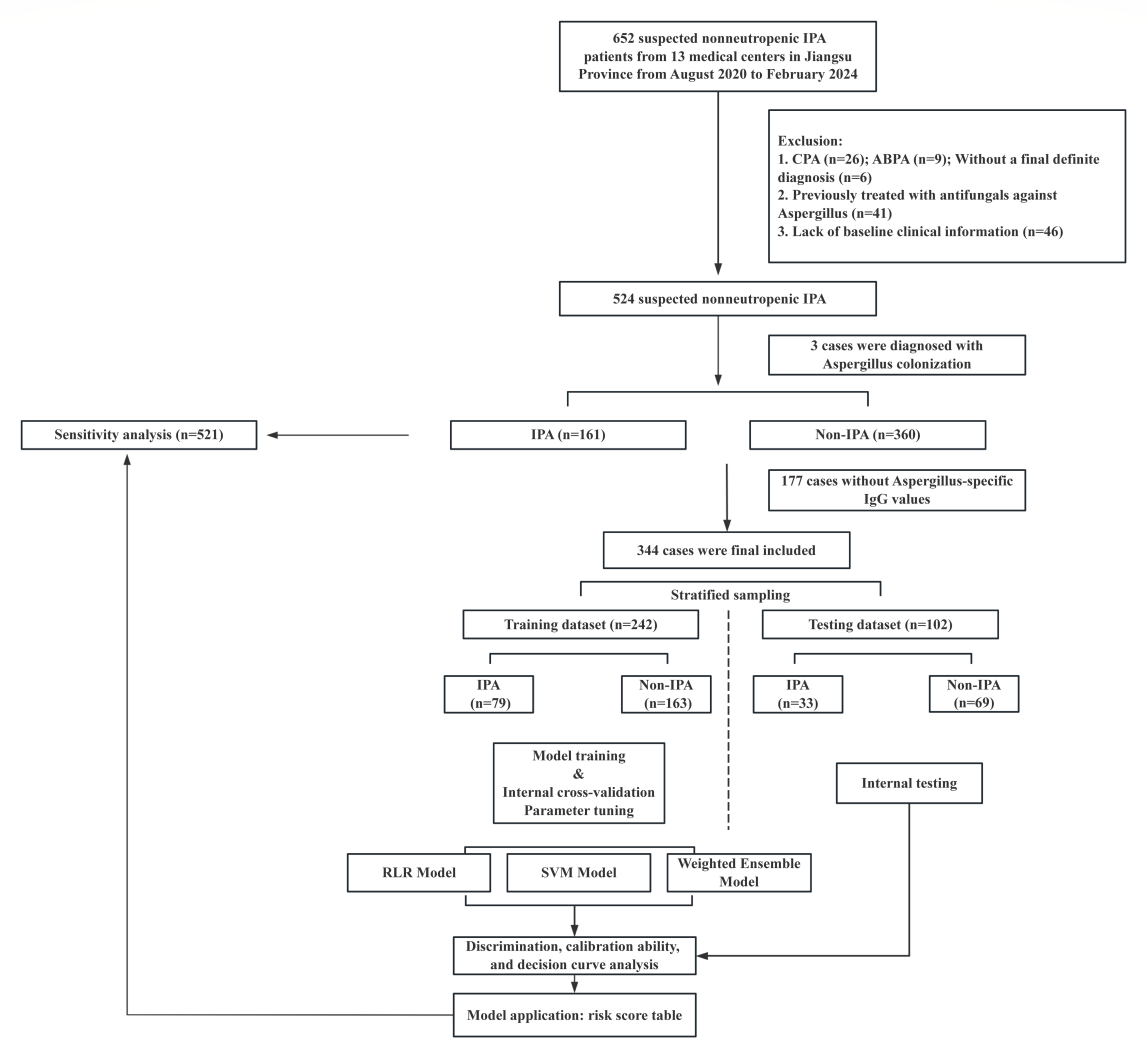
The flowchart of the study. Abbreviations: IPA, invasive pulmonary aspergillosis; CPA, chronic pulmonary aspergillosis; ABPA, allergic bronchopulmonary aspergillosis; RLR, regularized logistic regression; SVM, support vector machine.

A total of 344 patients with suspected nonneutropenic IPA from 13 medical centers in Jiangsu Province were enrolled in this study (Flowchart in Fig. 1). Of these, 112 were eventually diagnosed with IPA, including 5 proven cases, 102 probable cases, and 5 possible cases, with diagnostic evidence presented in Table S1. Non-IPA diagnoses included pulmonary infections due to other pathogens such as *Streptococcus pneumoniae*, *Staphylococcus aureus*, *Klebsiella pneumoniae*, *Pseudomonas aeruginosa*, tuberculosis, and non-tuberculous mycobacteria, as well as non-infectious causes like organizing pneumonia and lung cancer.

Stratified sampling was conducted based on the IPA diagnosis, and all subjects were divided into a training data set (242 cases, including 79 IPA and 163 Non-IPA) and a testing data set (102 cases, including 33 IPA and 69 Non-IPA). Characteristics of patients were shown in Table 1. Males accounted for 76.4% in training data set and 66.7% in testing data set. The median age was 67 years, and the average BMI was 21.92 kg/m². The most prevalent host factor was chronic pulmonary structural lesion (COPD, lung cancer, bronchiectasis, tuberculosis, or interstitial lung disease), accounting for approximately 60%. Other notable host factors included critical condition, systemic application of glucocorticoids, diabetes mellitus, and solid organ tumors (except lung cancer). Over 80% patients had cough or expectoration, and approximately half experienced fever, while hemoptysis was observed in less than 20% cases. No major differences in patient characteristics were observed between the training and testing sets.

**TABLE 1:** Characteristics of the training data set and testing data set^*a*^^,^^*b*^

	Overall (*n* = 344)	Training data set (*n* = 242)	Testing data set (*n* = 102)	*P*-value
IPA	112 (32.6)	79 (32.6)	33 (32.4)	
Non-IPA	232 (67.4)	163 (67.4)	69 (67.6)	
Age (median [IQR])	67.00 [57.00, 74.00]	66.50 [56.00, 73.00]	67.00 [59.00, 75.00]	0.060
Gender = Male (%)	253 (73.5)	185 (76.4)	68 (66.7)	0.060
BMI (mean [SD])	21.92 (3.58)	22.14 (3.62)	21.41 (3.43)	0.086
Smoking (%)	130 (37.8)	94 (38.8)	36 (35.3)	0.535
Host factors				
Critical condition (%)	124 (36.0)	83 (34.3)	41 (40.2)	0.298
COVID 2019/Influenza (%)	9 (2.6)	5 (2.1)	4 (3.9)	0.325
Chronic lung structural lesions (%)	201 (58.4)	141 (58.3)	60 (58.8)	0.923
Solid organ tumor (except lung cancer) (%)	49 (14.2)	32 (13.2)	17 (16.7)	0.404
Solid organ transplantation (%)	12 (3.5)	10 (4.1)	2 (2.0)	0.316
Diabetes (%)	83 (24.1)	62 (25.6)	21 (20.6)	0.319
Hepatopathy (%)	24 (7.0)	16 (6.6)	8 (7.8)	0.682
Chronic kidney disease (%)	39 (11.3)	30 (12.4)	9 (8.8)	0.340
Autoimmune disease (%)	37 (10.8)	30 (12.4)	7 (6.9)	0.130
Systemic application of glucocorticoids (%)	58 (16.9)	39 (16.1)	19 (18.6)	0.570
Application of immunosuppressants (%)	27 (7.8)	21 (8.7)	6 (5.9)	0.379
Clinical symptoms				
Fever (%)	164 (47.7)	115 (47.5)	49 (48.0)	0.930
Cough (%)	303 (88.1)	215 (88.8)	88 (86.3)	0.502
Sputum (%)	283 (82.3)	199 (82.2)	84 (82.4)	0.978
Hemoptysis (%)	67 (19.5)	48 (19.8)	19 (18.6)	0.796
Dyspnea (%)	170 (49.4)	118 (48.8)	52 (51.0)	0.707
Thoracalgia (%)	32 (9.3)	25 (10.3)	7 (6.9)	0.312
Chest CT features				
Halo or air crescent (%)	14 (4.1)	10 (4.1)	4 (3.9)	0.928
Nodule (%)	154 (44.8)	109 (45.0)	45 (44.1)	0.875
Cavity (%)	79 (23.0)	49 (20.2)	30 (29.4)	0.065
Pleural effusion (%)	115 (33.4)	83 (34.3)	32 (31.4)	0.599
Laboratory test				
Positive sputum aspergillus culture (%)	44 (12.8)	31 (12.8)	13 (12.7)	0.987
Serum GM (%)				0.878
<0.5	285 (82.8)	202 (83.5)	83 (81.4)	
0.5–1.0	30 (8.7)	20 (8.3)	10 (9.8)	
≥1.0	29 (8.4)	20 (8.3)	9 (8.8)	
Plasma PTX3, ng/mL (median [IQR])	3.84 [1.90, 8.05]	4.15 [1.92, 8.11]	3.12 [1.58, 7.54]	0.295
*Aspergillus*-specific IgG, AU/mL (median [IQR])	68.75 [40.49, 124.64]	66.46 [38.59, 118.01]	70.50 [48.13, 133.57]	0.306
C-reactive protein, mg/L (%)				0.523
<10	120 (34.9)	87 (36.0)	33 (32.4)	
≥10	224 (65.1)	155 (64.0)	69 (67.6)	
Procalcitonin, ng/mL (%)				0.128
<0.5	265 (77.0)	181 (74.8%)	84 (82.4)	
≥0.5	79 (23.0)	61 (25.2)	18 (17.6)	

^*a*^ Notes: Critical condition: admission to ICU, mechanical ventilation, severe pneumonia, sepsis/septic shock, or acute respiratory distress syndrome; chronic lung structural lesions: COPD, lung cancer, bronchiectasis, tuberculosis, or interstitial lung disease.

^*b*^ IPA: Invasive pulmonary aspergillosis; BMI: body mass index; GM: galactomannan; PTX3: Pentraxin 3; GM: galactomannan.

### Model training, internal cross-validation, and parameter tuning

**Fig 2:**
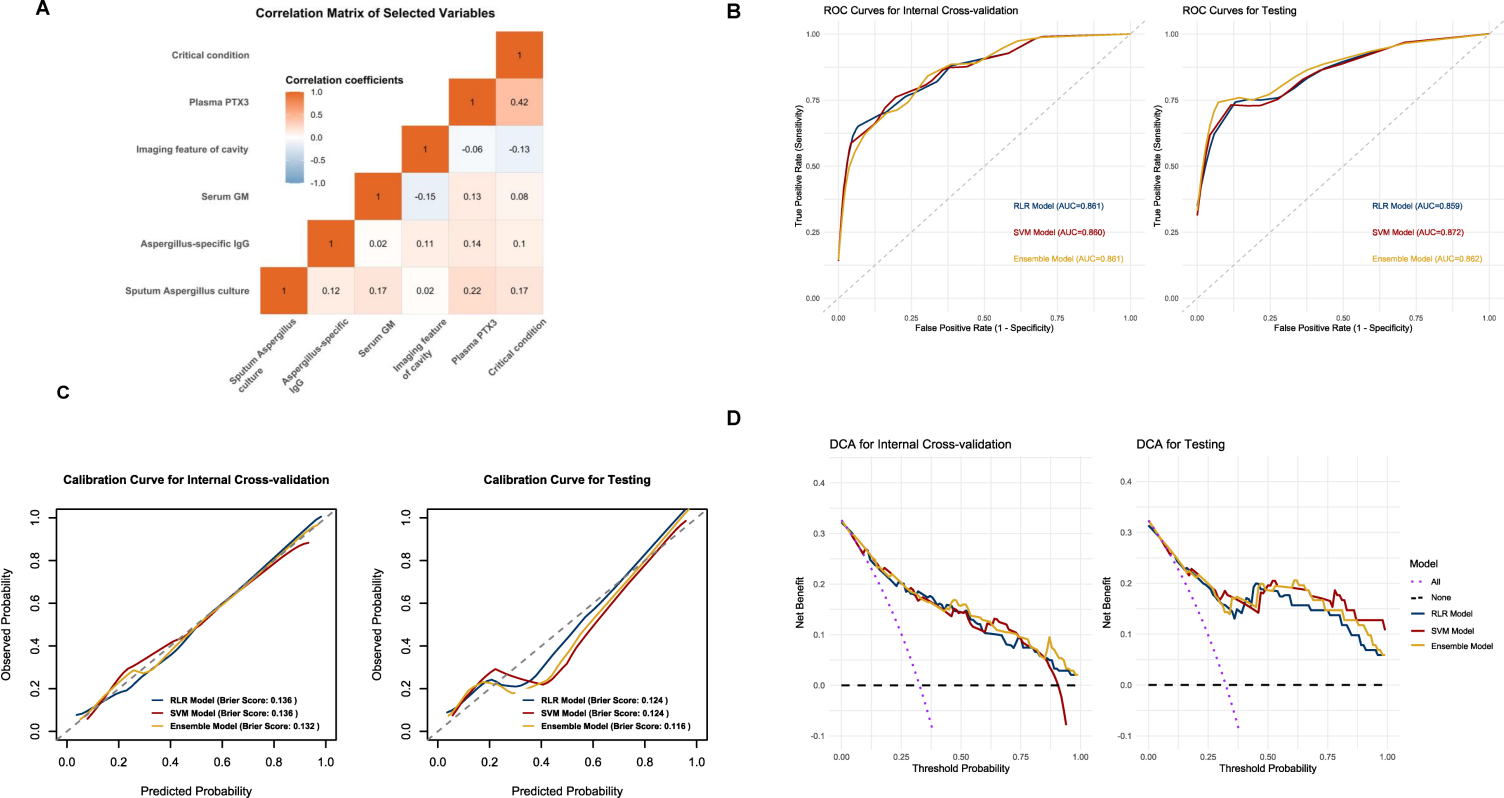
(**A**) Correlation analysis of selected variables; (**B**) receiver operating characteristic curve of the three models for diagnosing IPA in the internal cross-validation data set and the testing data set; (**C**) calibration curve of the three models in the internal cross-validation data set and the testing data set; (**D**) decision curve analysis of the three models in the internal cross-validation data set and the testing data set; Abbreviations: IPA, invasive pulmonary aspergillosis; RLR, regularized logistic regression; SVM, support vector machine; ROC, receiver operating characteristic; DCA, decision curve analysis.

Characteristics of patients grouped by IPA diagnosis in training data set were shown in Table S2. Inverse probability weighting was applied to weight the gender variable. First, potential variables were initially screened based on univariate analysis combined with Bonferroni correction (Bonferroni-corrected *P*-value < 0.05, Table S3). Subsequently, a stepwise regression optimization model using the BIC was adopted, reducing the BIC value from 347.5 to 330.6 (Table S4), significantly improving the balance between model complexity and goodness of fit. Finally, the stability of the variables was verified through Bootstrap resampling, and six core variables that repeatedly appeared in more than 80% of the samples were selected, including sputum *Aspergillus* culture, *Aspergillus*-specific IgG, imaging feature of cavity, serum GM, critical condition, and plasma PTX3 (Table S5). As depicted in Fig. 2A, no significant multicollinearity issue existed among the selected variables.

After variable selection, we developed an RLR model and an SVM model, optimizing the hyperparameters via three repeats of stratified fivefold cross-validation. The parameter combination selected was alpha = 0 and lambda = 0.06136 for the RLR model (Fig. S2), yielding a mean cross-validated AUC of 0.846 (range: 0.746–0.958; Table S6). For the SVM model, the best parameters combination was sigma = 0.03125 and *C* = 0.03125 (Fig. S3)**,** achieving a mean AUC of 0.837 (range: 0.789–0.911; Table S7). Furthermore, a weighted ensemble model was constructed with the predicted probabilities of both models given equal weight of 50% based on the AUC values.

### Model performance in internal cross-validation data set and testing data set

Model performance was evaluated based on discrimination, calibration, and clinical applicability. The ability of discrimination was quantified using AUC, sensitivity, specificity, and accuracy (Table 2). All three models have comparable AUC, exhibiting robust discriminative power, with AUC values consistently above 0.85 (Fig. 2B). Notably, at the optimal thresholds determined by the Youden index, the ensemble model achieved the significantly higher specificity of 95.1% (95% CI: 90.6–97.9) in the internal cross-validation data set and 95.7% (95% CI: 87.8–99.1) in the testing data set, indicating superior ability to correctly identify negative cases. The RLR model exhibited the relatively higher sensitivity of 73.4% (95% CI: 62.3–82.7) in internal cross-validation data set and 72.7% (95% CI: 54.5–86.7) in the testing data set, suggesting improved detection of true positive cases (Fig. S4).

**TABLE 2:** Discrimination of models in internal cross-validation data set and testing data set^*a*^^,^^*b*^

Data set	Sensitivity (%) (95% CI)	Specificity (%) (95% CI)	Accuracy (%) (95% CI)	AUC (95% CI)
RLR model				
Internal cross-validation	73.4% (62.3, 82.7)	84.0% (77.5, 89.3)	80.6% (75.0, 85.4)	0.861 (0.810–0.911)
Testing	72.7% (54.5, 86.7)	78.3% (66.7, 87.3)	76.5% (67.0, 84.3)	0.859 (0.775–0.944)
SVM model				
Internal cross-validation	70.9% (59.6, 80.6)	83.4% (76.8, 88.8)	79.3% (73.7, 84.3)	0.860 (0.811–0.910)
Testing	69.7% (51.3, 84.4)	85.5% (75.0, 92.8)	80.4% (71.4, 87.6)	0.872 (0.791–0.954)
Ensemble model				
Internal cross-validation	60.8% (49.1, 71.6)	95.1% (90.6, 97.9)	83.9% (78.6, 88.3)	0.861 (0.811–0.912)
Testing	69.7% (51.3, 84.4)	95.7% (87.8, 99.1)	87.3% (79.2, 93.0)	0.862 (0.777–0.947)

^*a*^ The optimal thresholds were determined based on the Youden index: 0.376 (RLR model), 0.402 (SVM model), and 0.468 (Ensemble model).

^*b*^ RLR, regularized logistic regression; SVM, support vector machine; CI, confidence interval; AUC, area under the curve.

Calibration was assessed by Brier score and calibration curve (Fig. 2C). The weighted ensemble model achieved a Brier score of 0.132 on the internal cross-validation data set, and a Brier score of 0.116 on the test data set, demonstrating the best calibration. Its calibration curve was closest to the ideal diagonal line (*y* = *x*), indicating better alignment between the predicted probabilities and the observed outcomes. However, all models showed poor calibration curves in the testing data set, particularly the SVM model, which could potentially be attributed to the limited sample size.

The clinical applicability was evaluated using DCA, which visualized the net benefits of the three models at various threshold probabilities (Fig. 2D). The net benefit arises from balancing the gains associated with true positives against the harms caused by false positives. This includes the clinical advantages of accurately identifying cases requiring intervention to prevent missed diagnoses, as well as the resource wastage and potential iatrogenic harm resulting from unnecessary interventions in low-risk cases. When the decision curve of the model consistently remains above the “All” and “None” reference lines within a specific threshold interval, it suggests that this model holds a distinct clinical net benefit advantage within this risk probability interval. Both in the internal cross-validation data set and testing data set, all three models exhibited obvious comparable net benefits within a specific threshold range.

### Model application

The weighted ensemble model, exhibiting the significantly higher specificity among the three models, was selected as the optimal prediction model despite comparable discrimination capacity, calibration ability, and clinical applicability across all models.

**Fig 3:**
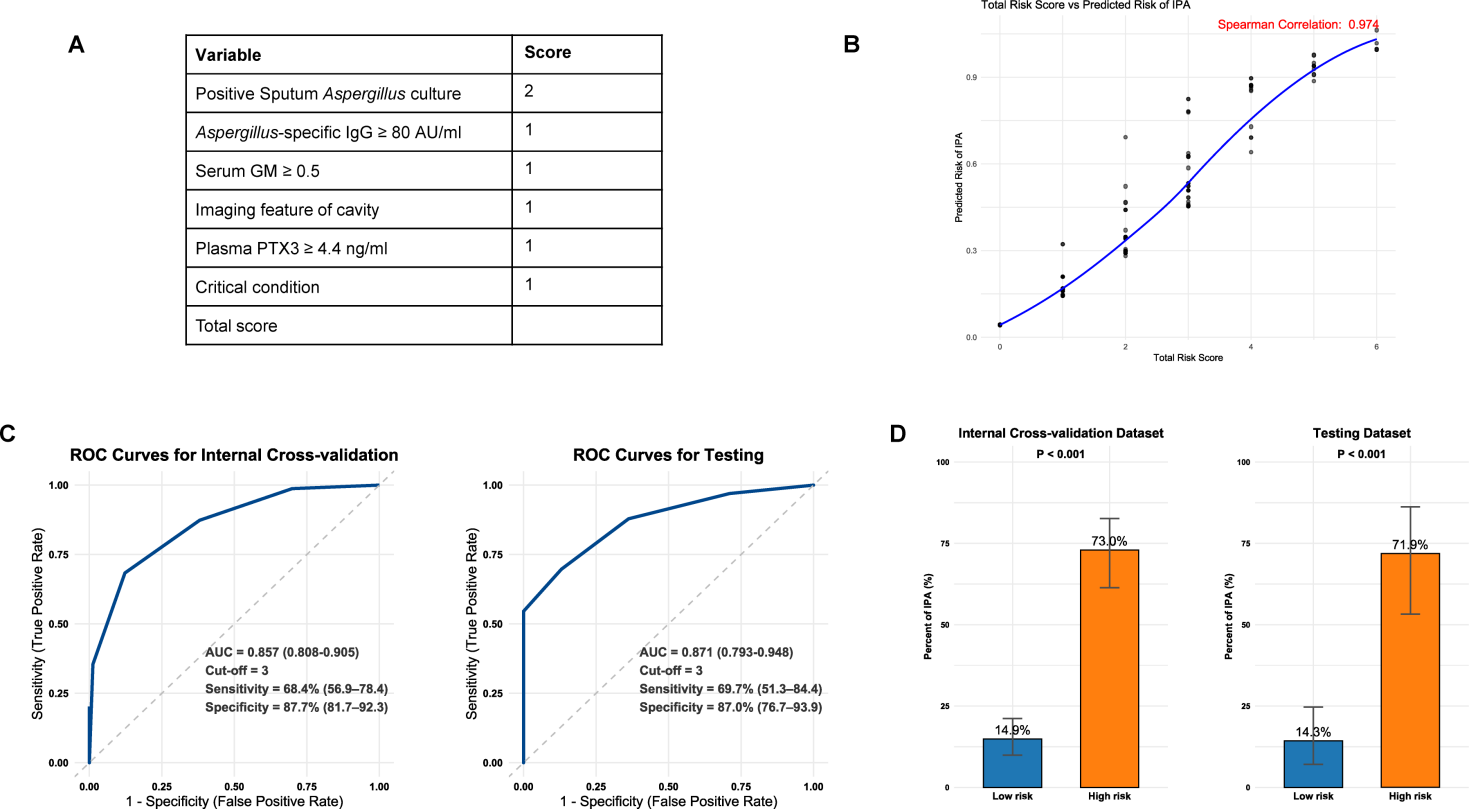
(**A**) Point values for each variable; Note: Serum GM 0.5–1.0 and ≥1.0 were both assigned a score of 1. (**B**) The correlation of total risk score with predicted IPA risk; (**C**) Receiver operating characteristic curve of the total score for diagnosing IPA; The best cut-off value is determined as three points using Youden index. (**D**) Comparison of IPA rate in low-risk (risk score <3) and high-risk (risk score ≥3) group. IPA, invasive pulmonary aspergillosis; ROC, receiver operating characteristic.

The importance of each predictor variable was weighted by the normalized SHAP values in the RLR model and the SVM model with a weight of 0.5 (Fig. S5 and Table S8). The minimum value was set as a reference and assigned a score of 1, with the remaining variables proportionally assigned (the score is assigned to the nearest integer) (Fig. 3A). The risk score correlated significantly with the predicted probability of the weighted ensemble model (Spearman *ρ* = 0.974) (Fig. 3B). The diagnostic performance of the risk score was evaluated using the ROC curve, confirming its robust discriminatory ability for IPA diagnosis. Specifically, the AUC values were 0.857 (95% CI: 0.808–0.905) for the internal cross-validation data set and 0.871 (95% CI: 0.793–0.948) for the testing data set (Fig. 3C). With an optimal cut-off value of 3, determined by the Youden index, the model exhibited high specificity. The specificity values were 87.7% (95% CI: 81.7–92.3) for the internal cross-validation data set and 87.0% (95% CI: 76.7–93.9) for the testing data set. As shown in Fig. 3D, clinical stratified analysis revealed that the rate of IPA in the high-risk group (score ≥3) was five times higher than that in the low-risk group (score <3) (*P* < 0.001). Notably, in the high-risk group, over 70% of patients were diagnosed with IPA, whereas in the low-risk group, over 85% of patients were confirmed as non-IPA cases. These findings suggest that this scoring system can facilitate rapid risk stratification of patients in clinical practice, offering an objective basis for early intervention in high-risk populations and rational treatment decisions for low-risk populations.

### Sensitivity analysis and subgroup analysis

Considering the potential for selection bias, we have conducted the sensitivity analysis. Sensitivity analysis validated the diagnostic performance of the risk score constructed based on the SHAP values in suspected IPA patients without excluding the missing values of *Aspergillus*-specific IgG (*n* = 521). The results indicated that the AUC of this risk score attained 0.869 (95% CI: 0.836–0.901), with a specificity of 87.8% (95% CI: 83.9–91.0) and a sensitivity of 68.3% (95% CI: 60.5–75.4) at the cut-off of 3 (Fig. S6A), comparable to the findings of the main study cohort (AUC of the internal cross-validation data set in the main cohort: 0.857; AUC of the testing data set: 0.871). Similarly, when conducting risk stratification based on a risk score threshold of 3, the confirmed rate of IPA in the high-risk group (71.4%) was significantly higher than that in the low-risk group (13.9%), with a risk ratio of 5 times (Fig. S6B), which was in accordance with the risk stratification trend of the main cohort. The consistency prediction performance between the two cohorts substantiated the diagnostic stability of this risk score under varying data integrity conditions.

Additionally, to evaluate the generalization ability of the risk score, we conducted stratified analyses in the total population and subgroups (age, gender, and underlying diseases). As illustrated in Fig. S7, the diagnosis rate of IPA in high-risk individuals (score ≥3) was significantly higher than that in low-risk individuals (score <3) in all subgroups (*P* < 0.001), especially in the patients younger than 65 years and the females.

### Compare the diagnostic performance of the established risk score with existing diagnostic criteria

The sensitivity and specificity in the total cohort (*n* = 344) of the established risk score and existing diagnostic criteria (*Aspergillus* culture, GM test, and Polymerase chain reaction [PCR]/metagenomic next-generation sequencing [mNGS]) were shown in Table S9. The total score established in this study exhibited a sensitivity equivalent to that of BALF GM (68.8% vs 61.3, *P* = 0.303), outperformed sputum *Aspergillus* culture, BALF *Aspergillus* culture, and serum GM (68.8% vs 40.2%, *P* < 0.001; 68.8% vs 30.9%, *P* < 0.001; 68.8% vs 16.5%, *P* < 0.001) but is inferior to PCR/mNGS (68.8% vs 82.7%, *P* = 0.040). The specificity of the total score was 87.5%, which was comparable to that of BALF GM and PCR/mNGS (87.5% vs 89.4%, *P* = 0.633; 87.5% vs 89.1%, *P* = 0.855), but lower than that of sputum *Aspergillus* culture, BALF *Aspergillus* culture, and serum GM (87.5% vs 98.9%, *P* < 0.001; 87.5% vs 99.3%, *P* < 0.001; 87.5% vs 95.0%, *P* = 0.007) (Fig. S8).

Additionally, Kaplan-Meier survival analysis in the IPA patients revealed significantly lower 30-day (log-rank *P* = 0.016) and 90-day (log-rank *P* = 0.038) survival rates in the high-risk group of risk score ≥3 compared to the low-risk group (Fig. S9). Cox regression analyses further supported the elevated risks of 30-day (HR: 3.952; 95% CI: 1.186–13.172; *P* = 0.025) and 90-day (HR: 2.665; 95% CI: 1.019–6.97; *P* = 0.046) mortality in the high-risk group of risk score ≥3 (Table S10), thereby underscoring the critical importance of early diagnosis, treatment, and intervention for high-risk patients based on the risk score.

## DISCUSSION

Nonneutropenic IPA has nonspecific clinical and radiographic features, making case identification challenging (12). Unlike previous researches, the study cohort focused on suspected nonneutropenic IPA cases with risk factors, abnormal CT features, and respiratory infections with failed treatment experience. And the incidence of IPA in the study was approximately one-third, higher than the 9.1%–9.6% reported in COPD populations (24, 25) and the 7.2%–19.2% observed in critically ill patients with influenza (26–28).

In this study, the machine learning models were established by integrating clinical characteristics, imaging features, and laboratory indicators, innovatively proposing a non-invasive and cost-effective diagnostic strategy for IPA diagnosis. The three models all demonstrated excellent discrimination (AUC > 0.85) and clinical applicability in both the internal cross-validation data set and the independent testing data set, particularly the weighted ensemble model. The risk table established based on the model SHAP values was highly consistent with the model predictions (*ρ* = 0.974), capable of significantly differentiating IPA from Non-IPA. The AUC values reached 0.857 in the internal cross-validation data set and 0.871 in the testing data set, and the robustness of the results was verified in subgroup analyses and sensitivity analyses. Compared with the indicators recommended in the current guidelines, this risk score (cut-off = 3) had a sensitivity of 68.8% comparable to that of BALF GM (61.3%), higher than that of serum GM, BALF *Aspergillus* culture, and sputum *Aspergillus* culture (16.6%, 30.9%, and 40.2% respectively), and was only surpassed by PCR/mNGS (82.7%). However, this risk score does not rely on invasive procedures to obtain BALF samples or on the higher-cost PCR/mNGS, thereby featuring higher clinical applicability. Although the specificity of the risk score was lower than that of *Aspergillus* cultures and serum GM, it still reached 87.5%, comparable to that of BALF GM and PCR/mNGS, and could effectively exclude low-risk individuals.

The diagnostic strategy incorporates the comprehensive effects of multiple variables, overcoming the limitation of the separated application. Our previous work has demonstrated the diagnostic value of *Aspergillus*-specific IgG and PTX3 in nonneutropenic IPA (13, 19). However, the increase of *Aspergillus* colonization and sensitization in patients with structural lung diseases may also lead to an increased level of *Aspergillus*-specific IgG (29, 30). Similarly, PTX3, an acute-phase inflammatory mediator produced and released at the infection site, is also linked to *Aspergillus* (31), but it lacks good specificity, associated with a variety of infections and inflammatory diseases, such as bacterial pneumonia and sepsis (32–34). Although sputum *Aspergillus* culture and serum GM test are less sensitive than BALF testing (13, 35), their specimens are relatively easy to obtain, making them valuable in clinical practice. The EORTC/MSGERC guideline recommends a positive threshold of 1.0 for the serum GM; however, this criterion is primarily based on data from neutropenic patients (7). Nonneutropenic IPA patients, however, retain partial neutrophil function and exhibit avascular invasion (36), which may lead to reduced GM release into the blood, thereby decreasing the sensitivity of the traditional threshold (37). This study demonstrates that a serum GM level of ≥0.5 already exhibits predictive value for diagnosing nonneutropenic IPA. Furthermore, the higher probability of diagnosing nonneutropenic IPA in critically ill patients and those with cavities appeared on the chest CT is consistent with previous findings (38, 39).

Apart from the novelty of the studied population and the integration of variables, this research has the following advancements. The study employed a weighted ensemble strategy to combine the strengths of the RLR and SVM models. The RLR model captures linear relationships, while the SVM model is suitable for complex, non-linear decision boundaries, and the weighted ensemble model enhances both discrimination and generalizability, while also improving robustness. Additionally, we employed internal cross-validation for parameter tuning. Through multiple rounds of training and validation, the approach maximizes the utilization of the available samples, reduces reliance on specific data partitioning, lowers the risk of overfitting, and enhances the generalization of the model (40, 41).

The study also had several limitations. First, the development and validation of the model were confined to patients suspected of having nonneutropenic IPA, which may limit its applicability in a broader population. Second, the poor performance of the model calibration curve in the testing data set may be attributed to the limited sample size and potential overfitting, thereby compromising the generalizability of the predicted probabilities. Future studies should prioritize expanding data sets to enhance probabilistic reliability. Moreover, the models did not incorporate the dynamic change of clinical features, which could affect the timeliness of identifying high-risk individuals.

The machine learning-based diagnostic model and risk score could assist in identifying nonneutropenic IPA from suspected cases with higher clinical applicability. However, several areas require improvement. Future research should focus on expanding the study population and incorporating dynamic clinical features to improve diagnostic accuracy and generalizability across diverse patient populations.

## Data Availability

The raw data supporting the findings of this study are available upon reasonable request by contact with the corresponding author.

## SUPPLEMENTAL MATERIAL

Supplemental material spectrum.00607-25-s0001.docx Fig. S1 to S9; Tables S1 to S10.

ASM does not own the copyrights to Supplemental Material that may be linked to, or accessed through, an article. The authors have granted ASM a non-exclusive, world-wide license to publish the Supplemental Material files. Please contact the corresponding author directly for reuse.
