# Supplementary material for: Development and validation of a machine learning-based diagnostic model for identifying nonneutropenic invasive pulmonary aspergillosis in suspected patients: a multicenter cohort study
Source: Microbiol Spectr. 2025 May 22;13(7):e00607-25. doi: 10.1128/spectrum.00607-25 (PMC12211027; doi:10.1128/spectrum.00607-25)
Supplement: Supplemental material — Fig. S1 to S9; Tables S1 to S10. [file spectrum.00607-25-s0001.docx]

**Development and Validation of A Machine Learning-Based Diagnostic Model for Identifying Nonneutropenic Invasive Pulmonary Aspergillosis in Suspected Patients: A Multicenter Cohort Study**

Xinyu Wang^#^, Yajie Lu^#^,Chao Sun, Huanhuan Zhong, Yuchen Cai, Min Cao, Xuefan Cui, Wenkui Sun, Li Wang, Xin Lu, Cheng Chen, Yanbin Chen, Chunlai Feng, Yujian Tao, Jun Zhou, Jiaxin Shi, Guoer Ma, Yuanqin Li, Xin Su*

Methods 2

Figure S1 5

Figure S2 6

Figure S3 7

Figure S4 8

Figure S5 9

Figure S6 10

Figure S7 11

Figure S8 12

Figure S9 13

Table S1 14

Table S2 15

Table S3 17

Table S4 18

Table S5 19

Table S6 20

Table S7 21

Table S8 22

Table S9 23

Table S10 24

# Methods

**Diagnosis of nonneutropenic invasive pulmonary aspergillosis (IPA)**

Proven IPA required one of the following: (a) Hyphae or melanized yeast-like forms identified from microscopic analysis in needle aspiration or biopsy specimen with tissue damage; (b) Positive culture result of a hyaline or pigmented mold from a sterile specimen consistent with infection site; (c) Amplification of fungal DNA by Polymerase Chain Reaction (PCR) combined with DNA sequencing in tissue; (d) Blood culture positive for mold in a compatible infectious process (usually contamination).

Based on the guidelines of the European Organization for Research and Treatment of Cancer and the Mycoses Study Group Education and Research Consortium (EORTC/MSGERC) updated in 2020, the probable IPA need meet the evidence of the host factor, clinical feature, and mycological evidence. The host factors were consistent with the inclusion criteria for suspected nonneutropenic IPA, proven to increase the risk of IPA. Clinical features of nonneutropenic IPA often more varied and lack specificity, with chest CT abnormalities including, but not limited to, dense lesions (with or without halo sign), air crescent sign, cavities, and consolidation. Mycologic evidence included: (a) Serum galactomannan (GM) test ≥ 1.0, bronchoalveolar lavage fluid (BALF) GM test ≥ 1.0, or BALF GM test ≥ 0.7 combined with BALF GM ≥ 0.8; (b) Positive *Aspergillus* PCR test; (c) Positive *Aspergillus* culture result of qualified specimen from sputum, BALF, bronchial brush, or aspirate. The diagnosis of possible IPA was made in cases where patients showed clinical signs and imaging findings consistent with IPA, but lacked definitive mycologic evidence.

The Invasive Fungal Diseases in Adult Patients in Intensive Care Unit (FUNDICU) 2024 consensus defined that a probable diagnosis required at least one symptom/sign, one ICU host factor, one clinical criterion, and one mycologic criterion, except for COVID-19 or influenza (without corresponding symptom/sign). Symptoms/signs: (a) Persistent fever after 3 days of appropriate antibiotics; (b) Fever recurrence 48 hours after defervescence while on antibiotics; (c) Pleuritic chest pain; (d) Pleural friction rub; (e) Dyspnea (only within the first 48 hours of ventilation); (f) Hemoptysis; (g) Worsening respiratory insufficiency despite treatment. ICU host factors: (a) Influenza; (b) COVID-19; (c) Moderate/severe COPD; (d) Decompensated liver cirrhosis; (e) Uncontrolled HIV (CD4 < 200/mm³); (f) Solid tumor. Clinical Criteria: (a) Bronchoscopy findings: ulcers, nodules, pseudo membranes, plaques, or crusts (probable TBA); (b) Chest computed tomography (CT): pulmonary infiltration or cavities not explained by other causes (probable IPA). Mycological Criteria: (a) Microscopic detection of fungal elements in BALF; (b) Positive *Aspergillus* culture in BALF; (c) Serum GM test > 0.5; (d) BALF GM test ≥ 1.0. Furthermore, the mycologic evidence of severe Chronic Obstructive Pulmonary Disease (COPD) patients underlined *Aspergillus* isolation from lower respiratory tract sample and two consecutive positive serum GM tests.

**Data collection**

To ensure consistency and high-quality data collection, standardized training was conducted at each research center before data collection began. Data collection, blood sampling, and assays were all performed without knowledge of the diagnostic results. Sputum Aspergillus culture, serum GM test, C-reactive protein and procalcitonin were routinely performed by the clinical center laboratories in each clinical center. Blood samples for the measurement of PTX3 (DPTX30, Quantizing Human Pentraxin 3 Immunoassay, R&D, Abingdon, UK) and Aspergillus-specific IgG (Dynamiker, Tianjin, China) were collected uniformly and tested using the enzyme-linked immunosorbent assay kit according to the protocol of manufacturer.

**Stratified** **sampling and dataset division**

In the process of dividing the dataset, an initial stratified sampling was conducted based on the outcome variable (IPA or Non-IPA) to ensure that the sample proportions across different categories remained consistent in both the training and testing dataset. Subsequently, the data was partitioned in a 7:3 ratio, with 70% allocated as the training dataset for model training and internal cross-validation, while the remaining 30% served as the testing dataset for model evaluation.

**Multiple imputation handling missing data**

Missing values were handled using Multiple Imputation by Chained Equations (MICE). Continuous variables were imputed via predictive mean matching to preserve the underlying data distribution by sampling observed values close to the predicted means. Binary variables were modeled using logistic regression, while multiclass categorical variables were modeled using multinomial regression, both generating probability-based imputations. Five imputed datasets (m=5) were generated to strike a balance between computational efficiency and statistical stability. Post-imputation analyses were combined using Rubin's rules, with continuous variables summarized by their means and categorical variables by their modes. Structural consistency was validated by comparing the distributions of variables before and after imputation.

**Inverse probability weighting to address gender imbalance**

To address the gender ratio imbalance in the sample, we adopted inverse probability weighting. This involved calculating the proportion of each gender in the training dataset and assigning weights accordingly, ensuring that each sample more evenly represented characteristics of both gender during training, thereby mitigating the impact of gender imbalance on the model.

**Variable selection**

The variable selection process followed a three-phase approach: initial screening via univariate analysis with Bonferroni correction (adjusted significance threshold α/k, where k = number of variables) to reduce false positives; subsequent optimization using bidirectional stepwise regression guided by the Bayesian Information Criterion (BIC) to balance model complexity and predictive accuracy; and final validation through Bootstrap resampling iterations, retaining variables consistently selected in ≥80% of subsamples to ensure robustness against data variability. Eventually, we employed the Spearman correlation coefficient (applicable for non-normal or ordinal variables) to compute the correlations among selected variables. Generally, a correlation coefficient with an absolute value greater than 0.7 is regarded as a high correlation, and at this point, the existence of multicollinearity should be considered.

**Model training, internal cross-****validation, and hyperparameter tuning**

Three machine learning models were developed and trained: a regularized logistic regression (RLR) model, a support vector machines (SVM) model, and a weighted ensemble model. RLR model is extensively employed in binary classification tasks, particularly effective for small datasets but maybe susceptible to overfitting. Hence, the RLR model was optimized over a hyperparameter grid covering Least Absolute Shrinkage and Selection Operator (LASSO), Ridge, and Elastic Net adopted, to enhance the generalization and robustness of the model. Model training and parameter optimization were accomplished through five-fold, three-repeated cross-validation. Regularization parameters were optimized via grid search over defined ranges: alpha (0 to 1) and lambda (10⁻⁴ to 10^0^). Alpha = 1 corresponded to Lasso (L1 regularization), alpha = 0 to Ridge (L2 regularization), and 0 < alpha < 1 represented Elastic Net. Lambda governed the regularization intensity with larger lambda values reducing model complexity by shrinking coefficients toward zero. The area under the curve (AUC) was used as the performance metric to select the optimal parameter combination, and random seed initialization ensured result reproducibility. Additionally, class imbalance was addressed with gender-specific weighting during model training.

The SVM model was trained and validated with the same cross-validation process, employing the radial basis function (RBF) kernel to capture non-linear relationships. Parameter tuning involved optimizing both the Sigma parameter of the kernel function and the penalty coefficient (C), with AUC guiding optimal parameter selection.

To capitalize on the complementary strengths of the two models, a weighted ensemble model was constructed using a weighted integration strategy. The RLR captures linear relationships well, while the SVM model handles complex, non-linear decision boundaries excellently. The final weighted ensemble model was generated based on AUC values of the two models, with weights assigned to each.

**Model evaluation and risk stratification**

We assessed the model performance on both the internal cross-validation dataset and the testing dataset, focusing on discrimination, calibration and clinical applicability. Discrimination was assessed using AUC, sensitivity, specificity, and accuracy. Calibration was evaluated through Brier score and calibration curves, and clinical applicability was analyzed using decision curve analysis (DCA). The optimal model was selected based on overall performance across these criteria, with the use of SHapley Additive exPlanation (SHAP) for interpretation. A risk score table was established based on the SHAP values. The correlation between the total risk score and the predicted probability was validated by Spearman analysis, suggesting that the scoring system could effectively mirror the model's prediction outcomes. The efficacy of the risk score for diagnosing IPA was determined through ROC analysis, and the optimal cut-off value for risk stratification was acquired. The robustness of the results was verified through sensitivity analysis and subgroup analysis, and the diagnostic capacity of the risk score was compared with the indicators recommended in the current guidelines.

# Figure S1


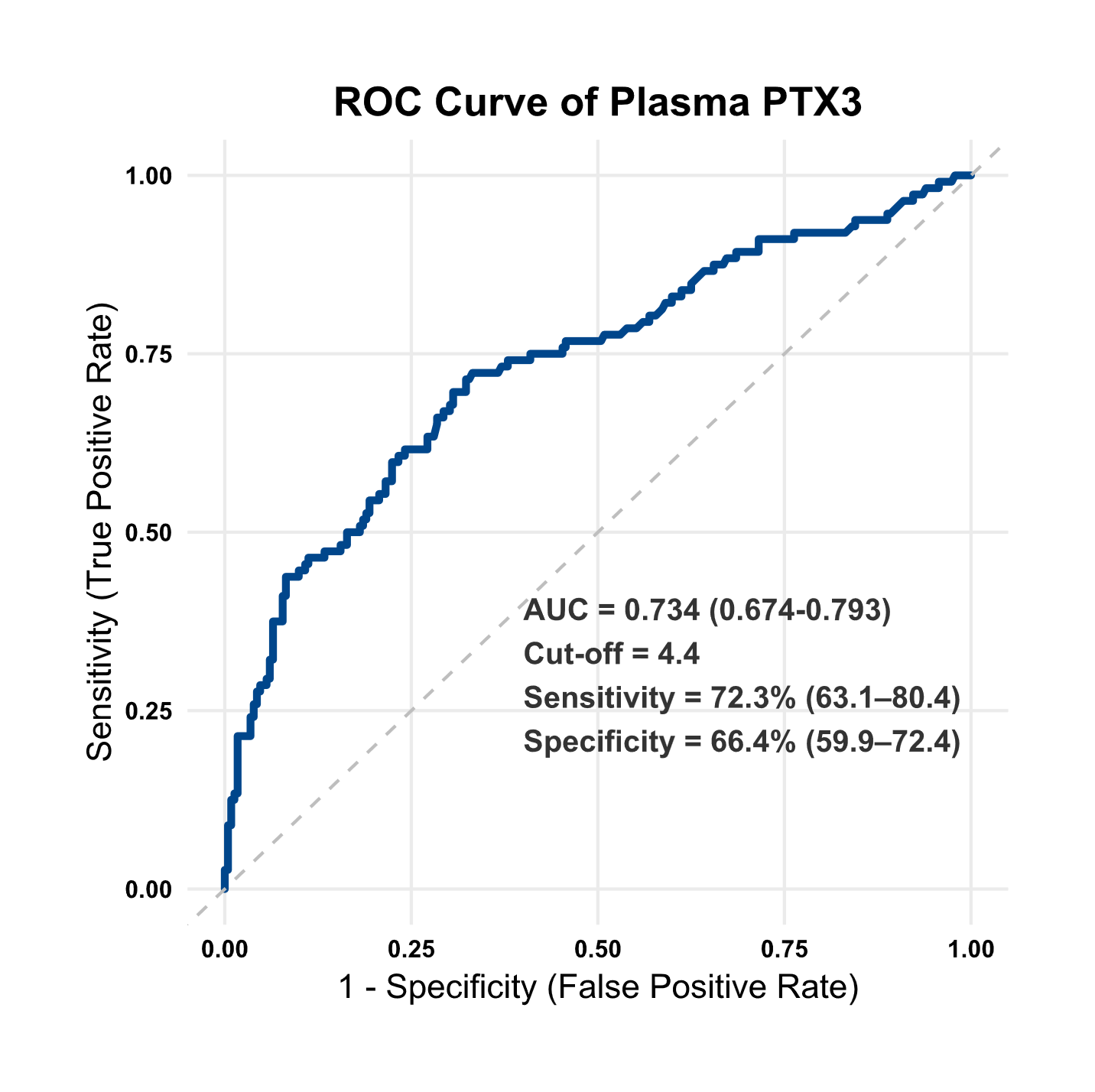


Figure legend: Receiver operating characteristic curve analysis of Plasma PTX3 for diagnosing IPA. The best cut-off value is determined as 4.4 ng/ml using Youden index. Abbreviations: ROC: receiver operating characteristic; AUC: area under the curve; PTX3: Pentraxin 3; IPA: invasive pulmonary aspergillosis.

# Figure S2


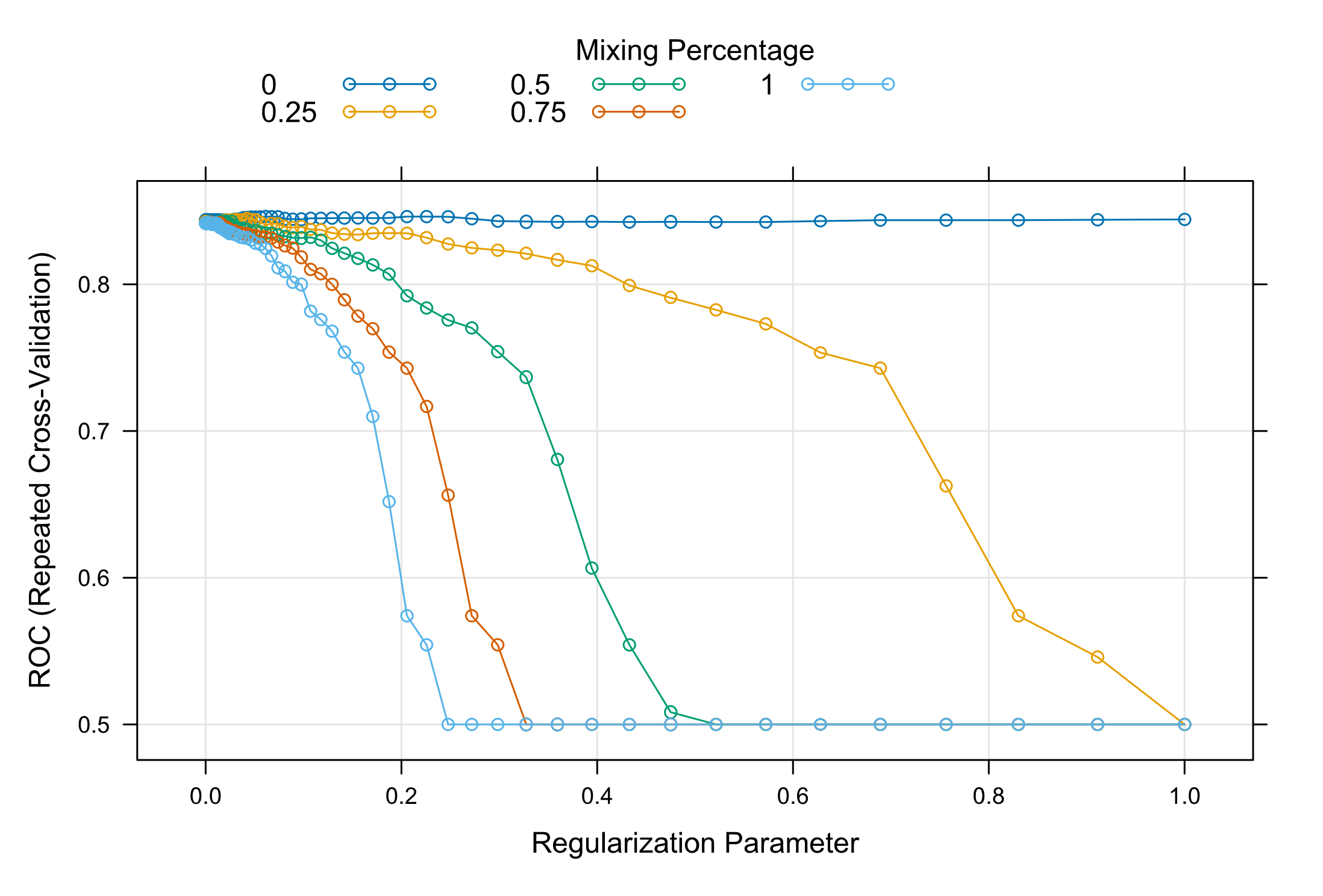


Figure legends: The parameter combination in regularized logistic regression model.

# Figure S3


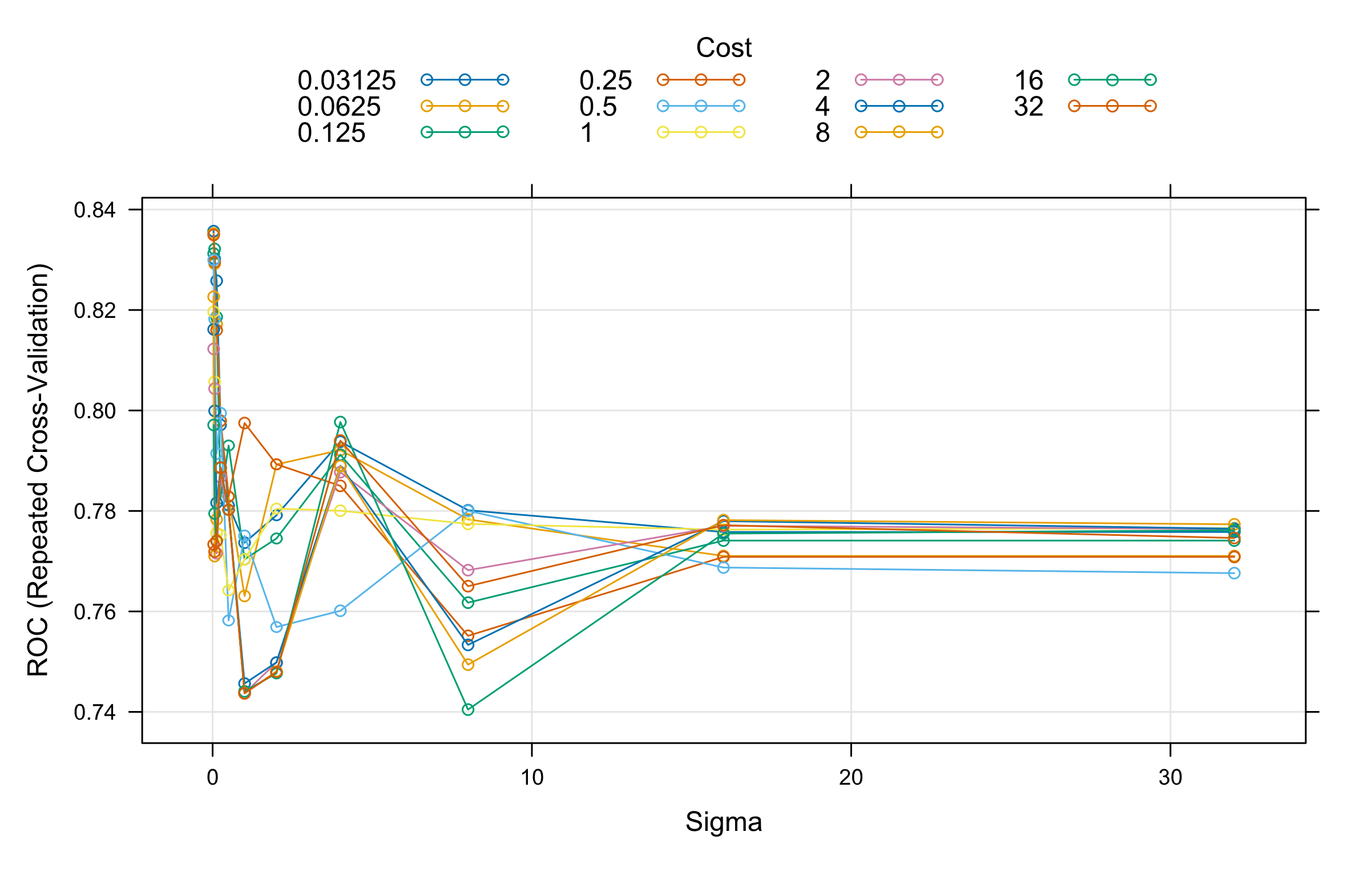


Figure legends: The parameter combination in support vector machine model.

# Figure S4


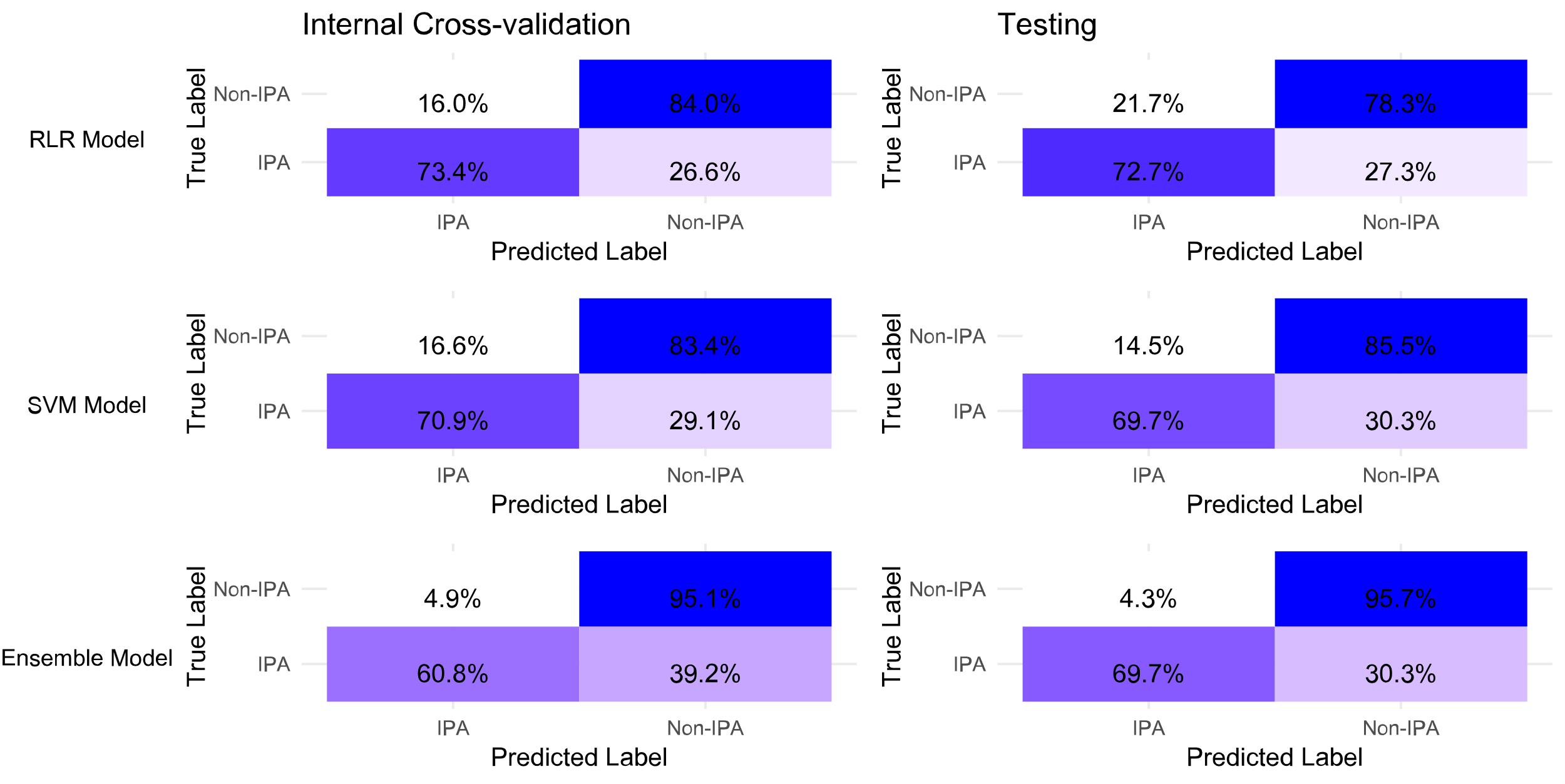


Figure legends: Confusion matrix of the regularized logistic regression model, support vector machine model, and ensemble model. Abbreviations: RLR: regularized logistic regression; SVM: support vector machine.

# Figure S5


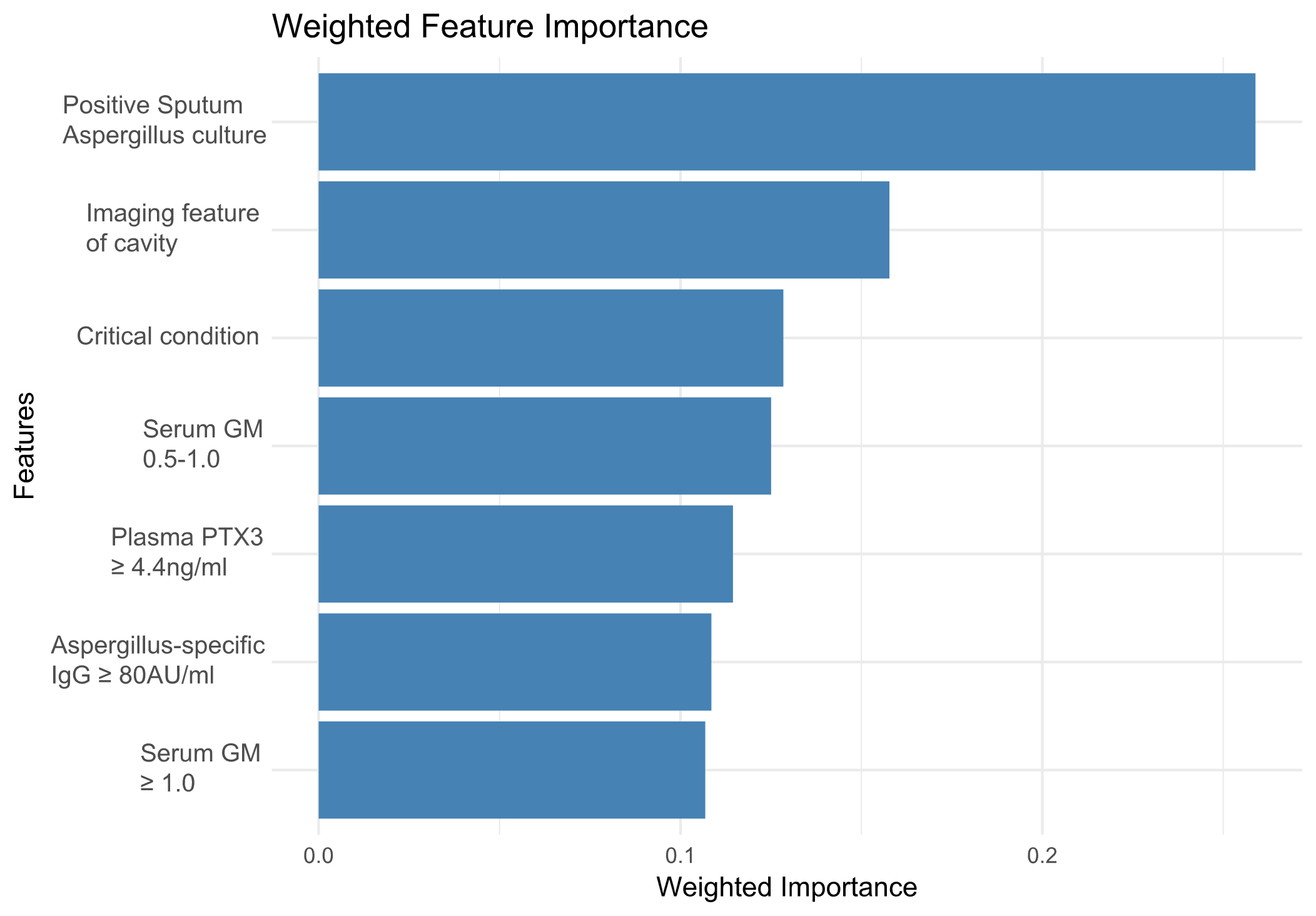


Figure legends: The weighted importance for each variable in weighted ensemble model. Abbreviations: GM: galactomannan; PTX3: pentraxin 3.

# Figure S6


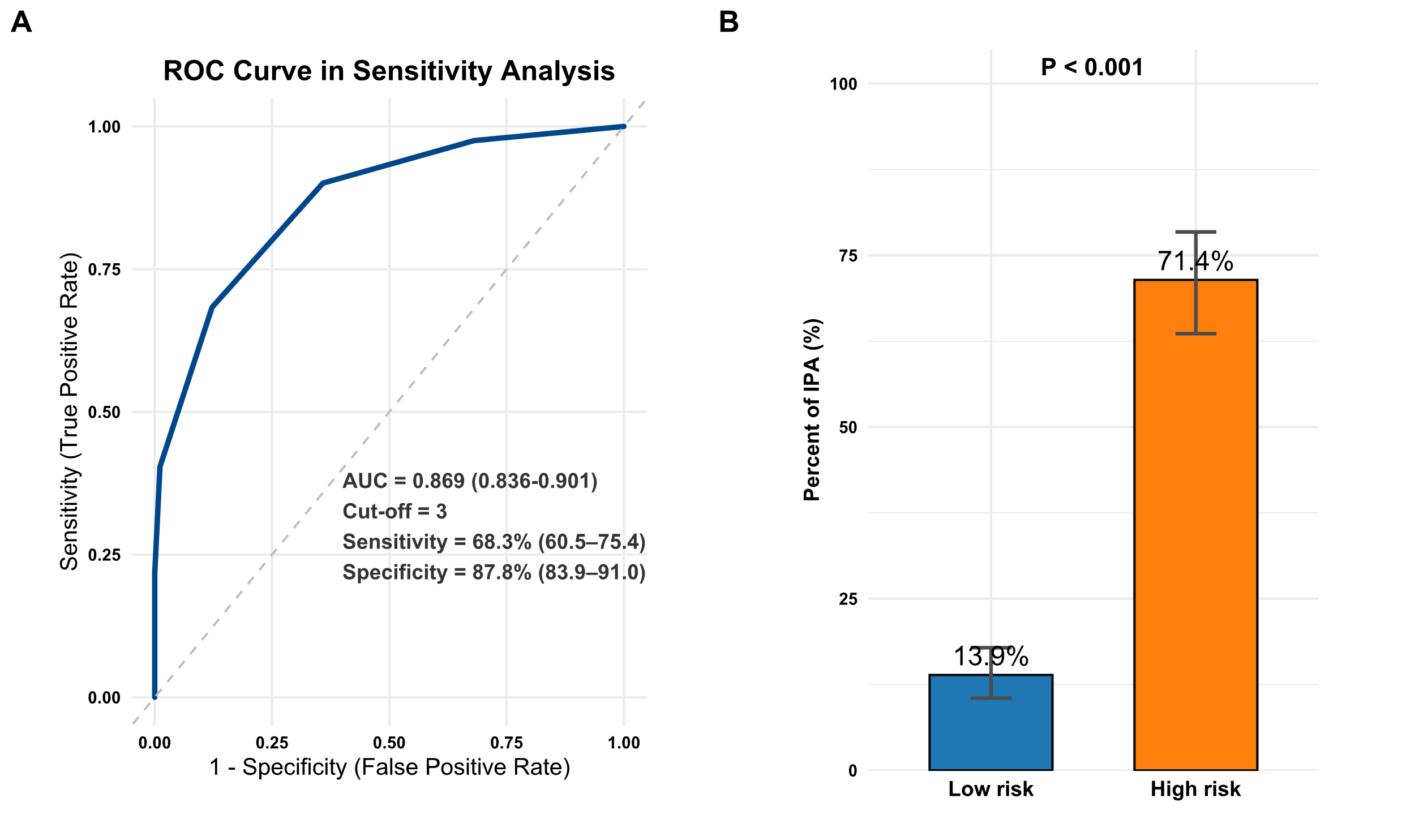


Figure legend: (A) Receiver operating characteristic curve of the risk score for diagnosing IPA in sensitivity analysis. (B) Comparison of IPA rate in low-risk (score < 3) and high-risk (score ≥ 3) group. Error bars represent 95% confidence intervals. Intergroup comparisons revealed statistically significant differences (P < 0.001). Abbreviation: ROC: receiver operating characteristic; AUC: area under the curve; IPA: invasive pulmonary aspergillosis.

# Figure S7


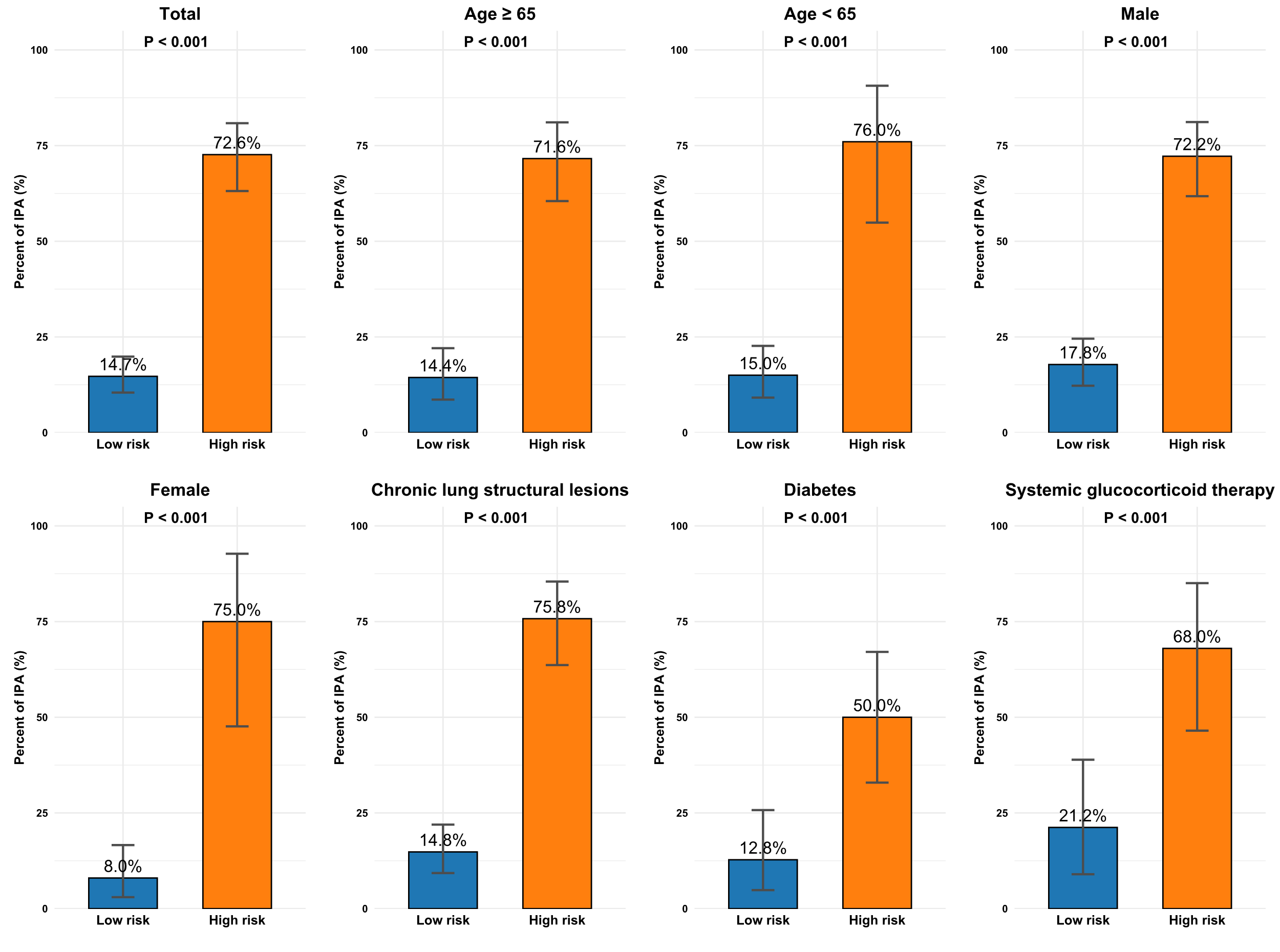


Figure legend: Subgroup analysis of risk stratification in suspected IPA patients

# Figure S8


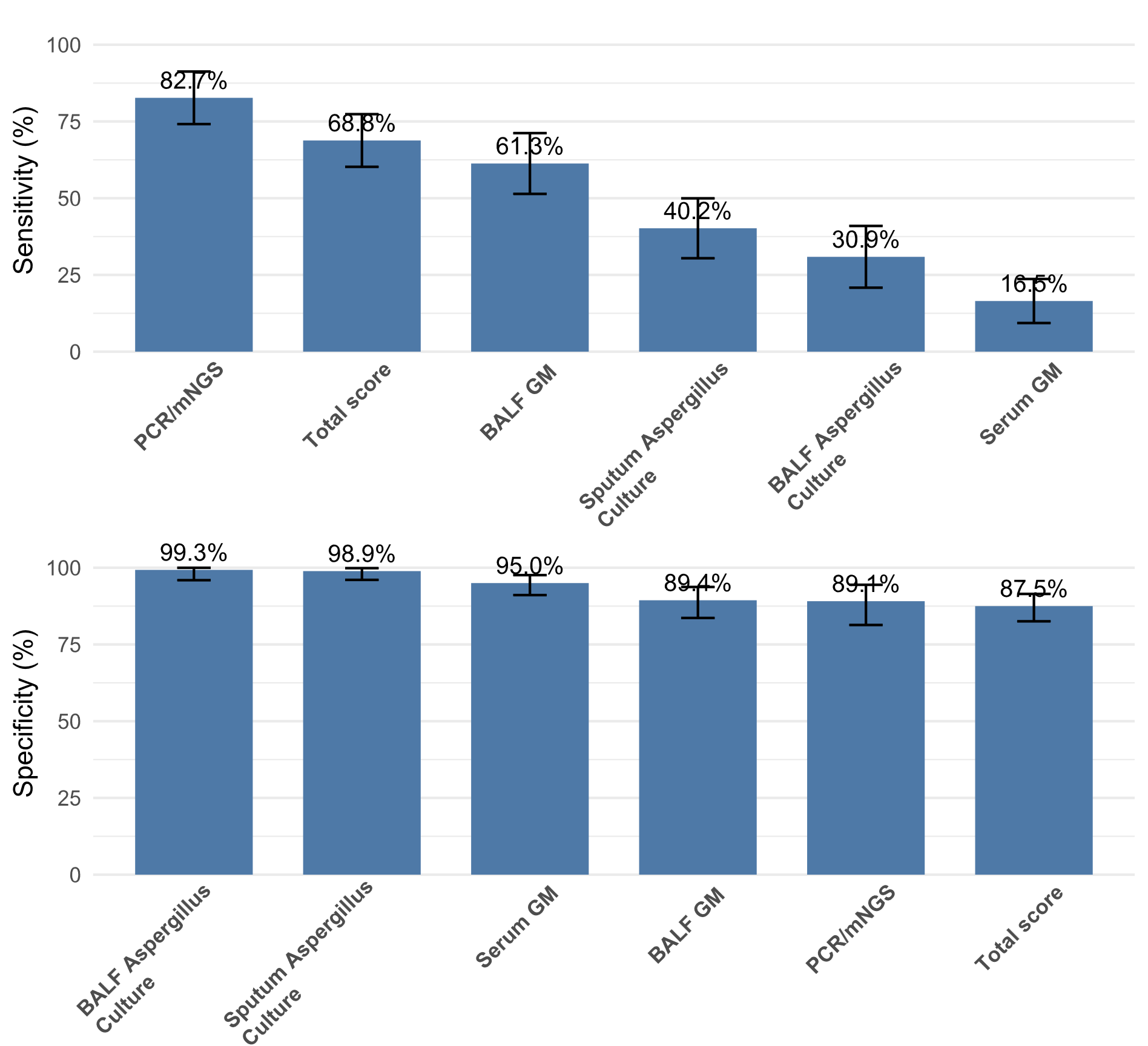


Figure legend: Comparison of the diagnostic performance between the established risk score and existing diagnostic criteria.

Sensitivity: Total score vs PCR/mNGS: 68.8% vs 82.7%, P = 0.040; Total score vs BALF GM: 68.8% vs 61.3, P = 0.303; Total score vs Sputum *Aspergillus* culture: 68.8% vs 40.2%, P < 0.001; Total score vs BALF *Aspergillus* culture: 68.8% vs 30.9%, P < 0.001; Total score vs serum GM: 68.8% vs 16.5%, P < 0.001. Specificity: Total score vs PCR/mNGS: 87.5% vs 89.1%, P = 0.855; Total score vs BALF GM: 87.5% vs 89.4%, P = 0.633; Total score vs serum GM: 87.5% vs 95.0%, P = 0.007; Total score vs Sputum *Aspergillus* culture: 87.5% vs 98.9%, P < 0.001; Total score vs BALF *Aspergillus* culture: 87.5% vs 99.3%, P < 0.001;

# Figure S9


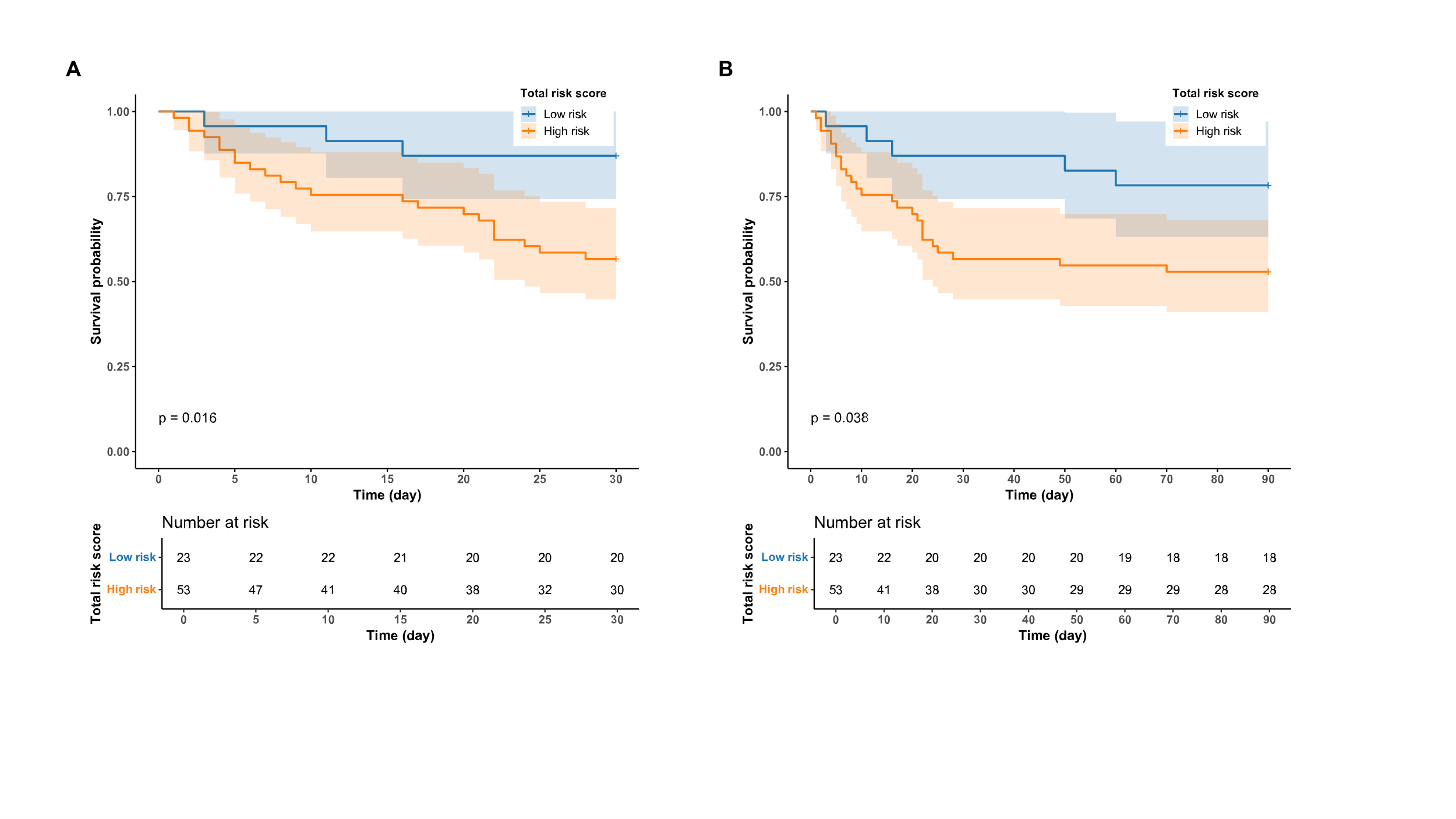


Figure legend: (A) Kaplan-Meier survival curve for 30-day survival based on risk score; log-rank P = 0.016; (B) Kaplan-Meier survival curve for 90-day survival based on risk score: log-rank P = 0.038. Low risk: risk score < 3; High risk: risk score ≥ 3.

# Table S1

**The evidence of IPA classifications.**

| IPA in total patients (IPA=112; Total=344) | Number |
| --- | --- |
| Proven IPA | **5** |
| Bronchoscopic biopsy | 3 |
| Lung puncture biopsy | 1 |
| Pleural fluid Aspergillus culture | 1 |
| Probable IPA | **102** |
| Sputum Aspergillus culture + BALF Aspergillus culture + Serum GM test ≥1.0 + BALF GM test ≥1.0 + Aspergillus mNGS/PCR | 2 |
| Sputum Aspergillus culture + BALF Aspergillus culture + Serum GM test ≥1.0 + BALF GM test ≥1.0 | 1 |
| Sputum Aspergillus culture + BALF Aspergillus culture + BALF GM test ≥1.0 + Aspergillus mNGS/PCR | 7 |
| Sputum Aspergillus culture + BALF Aspergillus culture + Serum GM test ≥1.0 + Aspergillus mNGS/PCR | 1 |
| Sputum Aspergillus culture + BALF Aspergillus culture + Serum GM test ≥1.0 | 1 |
| Sputum Aspergillus culture + BALF Aspergillus culture + BALF GM test ≥1.0 | 3 |
| Sputum Aspergillus culture + BALF Aspergillus culture + Aspergillus mNGS/PCR | 2 |
| Sputum Aspergillus culture + Serum GM test ≥1.0 + Aspergillus mNGS/PCR | 1 |
| Sputum Aspergillus culture + BALF GM test ≥1.0 + Aspergillus mNGS/PCR | 6 |
| BALF Aspergillus culture + BALF GM test ≥1.0 + Aspergillus mNGS/PCR | 4 |
| Serum GM test ≥1.0 + BALF GM test ≥1.0 + Aspergillus mNGS/PCR | 3 |
| Sputum Aspergillus culture + Serum GM test ≥1.0 | 3 |
| Sputum Aspergillus culture + BALF GM test ≥1.0 | 3 |
| Sputum Aspergillus culture + Aspergillus mNGS/PCR | 2 |
| Sputum Aspergillus culture + BALF Aspergillus culture | 2 |
| Serum GM test ≥1.0 + BALF GM test | 2 |
| Serum GM test ≥1.0 + Aspergillus mNGS/PCR | 1 |
| BALF GM test + BALF Aspergillus culture | 2 |
| BALF GM test + Aspergillus mNGS/PCR | 10 |
| BALF Aspergillus culture + Aspergillus mNGS/PCR | 2 |
| Sputum Aspergillus culture | 7 |
| Serum GM test ≥1.0 | 2 |
| BALF GM test ≥1.0 | 13 |
| Aspergillus mNGS/PCR | 21 |
| Critical condition + Serum GM test > 0.5^a^ | 1 |
| Possible IPA | **5** |

^a^ Although the case did not meet the “Probable” criteria according to the EORTC/MSGERC 2020, based on the definition in 2024 FUNDICU consensus, it was a “Probable IPA” (Serum GM test > 0.5).

Abbreviations: IPA: invasive pulmonary aspergillosis; BALF: Bronchoalveolar Lavage Fluid; GM: galactomannan; PCR: polymerase chain reaction; mNGS: metagenomic next-generation sequencing

# Table S2

Characteristics of patients in training dataset.

|  | Overall  (n=242) | IPA  (n=79) | Non-IPA  (n=163) | P-value |
| --- | --- | --- | --- | --- |
| Age ≥ 65 years (%) | 133 (55.0) | 53 (67.1) | 80 (49.1) | 0.008 |
| Sex = Male (%) | 185 (76.4) | 69 (87.3) | 116 (71.2) | 0.005 |
| BMI, kg/m2 (%) | |  |  | 0.276 |
| < 18.5 | 134 (55.4) | 49 (62.0) | 85 (52.1) |  |
| 18.5-24.0 | 34 (14.0) | 11 (13.9) | 23 (14.1) |  |
| ≥ 24.0 | 74 (30.6) | 19 (24.1) | 55 (33.7) |  |
| Smoking (%) | 94 (38.8) | 39 (49.4) | 55 (33.7) | 0.019 |
| Host factors |  |  |  |  |
| Critical condition (%) | 83 (34.3) | 43 (54.4) | 40 (24.5) | <0.001 |
| COVID 2019 / Influenza (%) | 5 (2.1) | 4 (5.1) | 1 (0.6) | 0.022 |
| Chronic lung structural lesions (%) | 141 (58.3) | 51 (64.6) | 90 (55.2) | 0.167 |
| Solid organ tumor (except lung cancer) (%) | 32 (13.2) | 13 (16.5) | 19 (11.7) | 0.301 |
| Solid organ transplantation (%) | 10 (4.1) | 1 (1.3) | 9 (5.5) | 0.119 |
| Diabetes (%) | 62 (25.6) | 17 (21.5) | 45 (27.6) | 0.309 |
| Hepatopathy (%) | 16 (6.6) | 4 (5.1) | 12 (7.4) | 0.500 |
| Chronic kidney disease (%) | 30 (12.4) | 10 (12.7) | 20 (12.3) | 0.932 |
| Autoimmune diseases (%) | 30 (12.4) | 9 (11.4) | 21 (12.9) | 0.741 |
| Systemic application of glucocorticoids (%) | 39 (16.1) | 16 (20.3) | 23 (14.1) | 0.223 |
| Application of immunosuppressants (%) | 21 (8.7) | 4 (5.1) | 17 (10.4) | 0.164 |
| Clinical Symptoms |  |  |  |  |
| Fever (%) | 115 (47.5) | 48 (60.8) | 67 (41.1) | 0.004 |
| Cough (%) | 215 (88.8) | 72 (91.1) | 143 (87.7) | 0.430 |
| Expectoration (%) | 199 (82.2) | 67 (84.8) | 132 (81.0) | 0.465 |
| Hemoptysis(%) | 48 (19.8) | 13 (16.5) | 35 (21.5) | 0.359 |
| Dyspnea (%) | 118 (48.8) | 50 (63.3) | 68 (41.7) | 0.002 |
| Thoracalgia (%) | 25 (10.3) | 9 (11.4) | 16 (9.8) | 0.706 |
| Chest CT features |  |  |  |  |
| Halo or air crescent (%) | 10 (4.1) | 7 (8.9) | 3 (1.8) | 0.010 |
| Nodule (%) | 109 (45.0) | 29 (36.7) | 80 (49.1) | 0.070 |
| Cavity (%) | 49 (20.2) | 24 (30.4) | 25 (15.3) | 0.006 |
| Pleural effusion (%) | 83 (34.3) | 39 (49.4) | 44 (27.0) | 0.001 |
| Laboratory test |  |  |  |  |
| Positive Sputum *Aspergillus* culture (%) | 31 (12.8) | 28 (35.4) | 3 (1.8) | <0.001 |
| Serum GM (%) | |  |  | 0.003 |
| <0.5 | 202 (83.5) | 57 (72.2) | 145 (89.0) |  |
| 0.5-1.0 | 20 (8.3) | 12 (15.2) | 8 (4.9) |  |
| ≥ 1.0 | 20 (8.3) | 10 (12.7) | 10 (6.1) |  |
| Plasma PTX3 ≥ 4.4 mg/ml (%) | 117 (48.3) | 57 (72.2) | 60 (36.8) | <0.001 |
| *Aspergillus*-specific IgG ≥ 80 AU/ml (%) | 97 (40.1) | 48 (60.8) | 49 (30.1) | <0.001 |
| C-reactive protein ≥ 10 mg/l (%) | 155 (64.0) | 60 (75.9) | 95 (58.3) | 0.007 |
| Procalcitonin ≥ 0.5 mg/ml (%) | 61 (25.2) | 24 (30.4) | 37 (22.7) | 0.197 |

Notes: When establishing the model, all continuous variables were transformed into categorical variables. In the absence of a recognized cut-off value, the optimal cut-off value of plasma PTX3 was determined to be 4.4 ng/ml through receiver operating characteristic curve analysis.

Critical condition: admission to ICU, mechanical ventilation, severe pneumonia, sepsis/septic shock, or acute respiratory distress syndrome; Chronic lung structural lesions: COPD, lung cancer, bronchiectasis, tuberculosis, or interstitial lung disease;

Abbreviations: IPA: Invasive pulmonary aspergillosis; BMI: body mass index; GM: galactomannan; PTX3: Pentraxin 3;

# Table S3

Univariate analysis with Bonferroni correction

| Variable | OR (95% CI) | P-value | Bonferroni-corrected  P-value | Significance |
| --- | --- | --- | --- | --- |
| Plasma PTX3 | 6.651 (4.210 - 10.507) | <0.001 | <0.001 | ** |
| Sputum *Aspergillus* culture | 28.792 (12.502 - 66.308) | <0.001 | <0.001 | ** |
| Critical condition | 3.955 (2.597 - 6.025) | <0.001 | <0.001 | ** |
| *Aspergillus*-specific IgG | 3.557 (2.346 - 5.392) | <0.001 | <0.001 | ** |
| Imaging feature of cavity | 3.594 (2.209 - 5.847) | <0.001 | <0.001 | ** |
| Imaging feature of pleural effusion | 2.982 (1.963 - 4.528) | <0.001 | <0.001 | ** |
| C-reactive protein | 3.138 (1.965 - 5.011) | <0.001 | <0.001 | ** |
| Gender | 2.796 (1.834 - 4.261) | <0.001 | <0.001 | ** |
| Fever | 2.446 (1.621 - 3.691) | <0.001 | <0.001 | ** |
| Dyspnea | 2.139 (1.415 - 3.234) | <0.001 | 0.010 | ** |
| Serum GM | 3.196 (1.693 - 6.033) | <0.001 | 0.010 | ** |
| Imaging feature of halo or air crescent | 5.033 (1.928 - 13.136) | <0.001 | 0.030 | ** |
| Smoking | 1.996 (1.302 - 3.061) | 0.002 | 0.047 | ** |
| Hemoptysis | 0.447 (0.254 - 0.786) | 0.005 | 0.159 | * |
| Age | 1.746 (1.162 - 2.623) | 0.007 | 0.226 | * |
| Solid organ tumor (except lung cancer) | 2.011 (1.192 - 3.392) | 0.009 | 0.274 | * |
| COVID 2019 / Influenza | 10.981 (1.599 - 75.402) | 0.015 | 0.458 | * |
| Procalcitonin | 1.702 (1.103 - 2.626) | 0.016 | 0.503 | * |
| BMI | 0.979 (0.547 - 1.755) | 0.944 | 1.000 |  |
| Chronic lung structural lesions | 0.824 (0.551 - 1.232) | 0.346 | 1.000 |  |
| Solid organ transplantation | 0.188 (0.032 - 1.124) | 0.067 | 1.000 |  |
| Diabetes | 1.097 (0.682 - 1.765) | 0.702 | 1.000 |  |
| Hepatopathy | 1.171 (0.502 - 2.734) | 0.715 | 1.000 |  |
| Chronic kidney disease | 0.904 (0.466 - 1.754) | 0.765 | 1.000 |  |
| Autoimmune diseases | 0.542 (0.296 - 0.993) | 0.047 | 1.000 | * |
| Systemic application of glucocorticoids | 1.502 (0.895 - 2.521) | 0.124 | 1.000 |  |
| Application of immunosuppressants | 0.714 (0.355 - 1.433) | 0.343 | 1.000 |  |
| Cough | 1.207 (0.611 - 2.383) | 0.589 | 1.000 |  |
| Expectoration | 1.023 (0.596 - 1.755) | 0.935 | 1.000 |  |
| Thoracalgia | 1.495 (0.807 - 2.771) | 0.201 | 1.000 |  |
| Imagine feature of nodule | 0.643 (0.427 - 0.969) | 0.035 | 1.000 | * |

** P-value < 0.05 & Bonferroni-corrected P-value < 0.05

* Only P-value < 0.05

Variables with a Bonferroni-corrected P-value < 0.05 were further subjected to stepwise regression, including plasma PTX3, sputum *Aspergillus* culture, critical condition, *Aspergillus*-specific IgG, imaging feature of cavity, imaging feature of pleural effusion, C-reactive protein, gender, fever, dyspnea, serum GM, imaging, feature of halo or air crescent, and smoking.

# Table S4

Results of stepwise regression

| Variable | β | OR (95% CI) | P-value |
| --- | --- | --- | --- |
| Plasma PTX3 ≥ 4.4 ng/ml | 1.367 | 3.923 (2.168-7.277) | <0.001 |
| Positive Sputum Aspergillus culture | 2.796 | 16.379 (6.733-45.541) | <0.001 |
| Critical condition | 1.342 | 3.827 (2.064-7.272) | <0.001 |
| *Aspergillus*-specific IgG ≥ 80 AU/ml | 1.088 | 2.969 (1.679-5.327) | <0.001 |
| Imaging feature of cavity | 1.778 | 5.918 (2.916-12.337) | <0.001 |
| Imaging feature of pleural effusion | 0.787 | 2.197 (1.180-4.114) | 0.013 |
| Serum GM 0.5-1.0 | 1.532 | 4.625 (1.896-11.427) | <0.001 |
| Serum GM ≥ 1.0 1.0 | 1.158 | 3.183 (0.958-10.193) | 0.054 |
| Imaging feature of halo or air crescent | 1.530 | 4.619 (1.362-15.948) | 0.014 |
| Smoking | 0.793 | 2.21 (1.221-4.031) | 0.009 |
| BIC =330.6 |  |  |  |

Abbreviations: GM: galactomannan; PTX3: Pentraxin 3; BIC: Bayesian Information Criterion.

# Table S5

Results of Bootstrap stability test

| Variable | Selected (%) |
| --- | --- |
| Sputum *Aspergillus* culture | 100.0 |
| *Aspergillus*-specific IgG | 98.5 |
| Imaging feature of cavity | 93.5 |
| Serum GM | 91.0 |
| Critical condition | 89.0 |
| Plasma PTX3 | 88.5 |
| Imaging feature of halo or air crescent | 76.0 |
| Smoking | 74.5 |
| Imaging feature of pleural effusion | 71.0 |

Six core variables that repeatedly appeared in more than 80% of the samples were selected, including sputum *Aspergillus* culture, *Aspergillus*-specific IgG, imaging feature of cavity, serum GM, and critical condition, and plasma PTX3

# Table S6

The AUC values of each fold in cross-validation in regularized logistic regression model

| Resample | AUC |
| --- | --- |
| Fold1.Rep1 | 0.901 |
| Fold1.Rep2 | 0.862 |
| Fold1.Rep3 | 0.826 |
| Fold2.Rep1 | 0.809 |
| Fold2.Rep2 | 0.861 |
| Fold2.Rep3 | 0.958 |
| Fold3.Rep1 | 0.795 |
| Fold3.Rep2 | 0.922 |
| Fold3.Rep3 | 0.746 |
| Fold4.Rep1 | 0.900 |
| Fold4.Rep2 | 0.783 |
| Fold4.Rep3 | 0.937 |
| Fold5.Rep1 | 0.823 |
| Fold5.Rep2 | 0.804 |
| Fold5.Rep3 | 0.767 |

# Table S7

The AUC values of each fold in cross-validation in support vector machine model

| Resample | AUC |
| --- | --- |
| Fold1.Rep1 | 0.872 |
| Fold1.Rep2 | 0.791 |
| Fold1.Rep3 | 0.791 |
| Fold2.Rep1 | 0.828 |
| Fold2.Rep2 | 0.789 |
| Fold2.Rep3 | 0.799 |
| Fold3.Rep1 | 0.849 |
| Fold3.Rep2 | 0.911 |
| Fold3.Rep3 | 0.800 |
| Fold4.Rep1 | 0.848 |
| Fold4.Rep2 | 0.864 |
| Fold4.Rep3 | 0.874 |
| Fold5.Rep1 | 0.834 |
| Fold5.Rep2 | 0.793 |
| Fold5.Rep3 | 0.892 |

# Table S8

Importance of variables

| Variable | SHAP-RLR | SHAP-SVM | Weighted Importance | Score |
| --- | --- | --- | --- | --- |
| Positive Sputum Aspergillus culture | 0.311 | 0.203 | 0.257 | 2 |
| Aspergillus-specific IgG ≥ 80 AU/ml | 0.091 | 0.124 | 0.107 | 1 |
| Serum GM 0.5-1.0 | 0.111 | 0.142 | 0.126 | 1 |
| Serum GM ≥ 1.0 | 0.082 | 0.132 | 0.107 | 1 |
| Imaging feature of cavity | 0.176 | 0.134 | 0.155 | 1 |
| Plasma PTX3 ≥ 4.4 ng/ml | 0.108 | 0.126 | 0.117 | 1 |
| Critical condition | 0.120 | 0.139 | 0.130 | 1 |

The importance of each predictor variable was weighted by the normalized SHAP values in the RLR model and the SVM model with a weight of 0.5. Abbreviations: RLR: regularized logistic regression; SVM: support vector machine; BALF: bronchoalveolar lavage fluid; GM: galactomannan; PTX3: Pentraxin 3.

# Table S9

The diagnostic performance of the established risk score and existing diagnostic criteria

| Test | TP | FN | FP | TN | Sensitivity | Specificity |
| --- | --- | --- | --- | --- | --- | --- |
| Sputum Aspergillus culture | 39 | 58 | 2 | 178 | 40.2% (30.4–50.7) | 98.9% (96.0–99.9) |
| BALF Aspergillus culture | 25 | 56 | 1 | 134 | 30.9% (21.1–42.1) | 99.3% (95.9–100.0) |
| Serum GM | 17 | 86 | 10 | 192 | 16.5% (9.9–25.1) | 95.0% (91.1–97.6) |
| BALF GM | 57 | 36 | 17 | 144 | 61.3% (50.6–71.2) | 89.4% (83.6–93.7) |
| PCR/mNGS | 62 | 13 | 11 | 90 | 82.7% (72.2–90.4) | 89.1% (81.3–94.4) |
| Total score | 77 | 35 | 29 | 203 | 68.8% (59.3–77.2) | 87.5% (82.5–91.5) |

Analyses were performed in total cohort (n=344)

Serum GM: cut-off = 1.0; BALF GM: cut-off =1.0; Total score: cut-off = 3

Abbreviations: BALF: bronchoalveolar lavage fluid; GM: galactomannan; PCR: polymerase chain reaction; mNGS: metagenomic next-generation sequencing.

# Table S10

Results of cox regression analysis for association of risk score with 30-day mortality and 90-day mortality

|  | **Survival** | **Non-Survival** | **HR (95% CI)** | **P-value** |
| --- | --- | --- | --- | --- |
| **30-day mortality** | |  |  |  |
| Low risk (risk score < 3) | 20 (40.0%) | 3 (11.5%) | ref |  |
| High risk (risk score ≥ 3) | 30 (60.0%) | 23 (88.5%) | 3.952 (1.186-13.172) | 0.025 |
| **90-day mortality** | |  |  |  |
| Low risk (score < 3) | 18 (39.1%) | 5 (16.7%) | ref |  |
| High risk (score ≥ 3) | 28 (60.9%) | 25 (83.3%) | 2.665 (1.019-6.97) | 0.046 |
